# The Stroke Recovery in Motion Implementation Planner: Mixed Methods User Evaluation

**DOI:** 10.2196/37189

**Published:** 2022-07-29

**Authors:** Jessica Reszel, Joan van den Hoek, Tram Nguyen, Gayatri Aravind, Mark T Bayley, Marie-Louise Bird, Kate Edwards, Janice J Eng, Jennifer L Moore, Michelle L A Nelson, Michelle Ploughman, Julie Richardson, Nancy M Salbach, Ada Tang, Ian D Graham

**Affiliations:** 1 Ottawa Hospital Research Institute Ottawa, ON Canada; 2 School of Nursing University of Ottawa Ottawa, ON Canada; 3 School of Epidemiology and Public Health University of Ottawa Ottawa, ON Canada; 4 March of Dimes Canada Toronto, ON Canada; 5 Division of Physical Medicine and Rehabilitation University of Toronto Toronto, ON Canada; 6 The KITE Research Institute University Health Network Toronto, ON Canada; 7 College of Health and Medicine University of Tasmania Tasmania Australia; 8 Department of Physical Therapy University of British Columbia Vancouver, BC Canada; 9 South Eastern Norway Regional Knowledge Translation Center Sunnaas Rehabilitation Hospital Oslo Norway; 10 Institute for Knowledge Translation Carmel, IN United States; 11 Lunenfeld-Tanenbaum Research Institute Sinai Health Toronto, ON Canada; 12 Dalla Lana School of Public Health University of Toronto Toronto, ON Canada; 13 Faculty of Medicine Memorial University of Newfoundland St John's, NL Canada; 14 School of Rehabilitation Science McMaster University Hamilton, ON Canada; 15 Department of Physical Therapy University of Toronto Toronto, ON Canada

**Keywords:** stroke, rehabilitation, community-based exercise programs, knowledge translation, knowledge mobilization, implementation science

## Abstract

**Background:**

As more people are surviving stroke, there is a growing need for services and programs that support the long-term needs of people living with the effects of stroke. Exercise has many benefits; however, most people with stroke do not have access to specialized exercise programs that meet their needs in their communities. To catalyze the implementation of these programs, our team developed the Stroke Recovery in Motion Implementation Planner, an evidence-informed implementation guide for teams planning a community-based exercise program for people with stroke.

**Objective:**

This study aimed to conduct a user evaluation to elicit user perceptions of the usefulness and acceptability of the Planner to inform revisions.

**Methods:**

This mixed methods study used a concurrent triangulation design. We used purposive sampling to enroll a diverse sample of end users (program managers and coordinators, rehabilitation health partners, and fitness professionals) from three main groups: those who are currently planning a program, those who intend to plan a program in the future, and those who had previously planned a program. Participants reviewed the Planner and completed a questionnaire and interviews to identify positive features, areas of improvement, value, and feasibility. We used descriptive statistics for quantitative data and content analysis for qualitative data. We triangulated the data sources to identify Planner modifications.

**Results:**

A total of 39 people participated in this study. Overall, the feedback was positive, highlighting the value of the Planner’s comprehensiveness, tools and templates, and real-world examples. The identified areas for improvement included clarifying the need for specific steps, refining navigation, and creating more action-oriented content. Most participants reported an increase in knowledge and confidence after reading the Planner and reported that using the resource would improve their planning approach.

**Conclusions:**

We used a rigorous and user-centered process to develop and evaluate the Planner. End users indicated that it is a valuable resource and identified specific changes for improvement. The Planner was subsequently updated and is now publicly available for community planning teams to use in the planning and delivery of evidence-informed, sustainable, community-based exercise programs for people with stroke.

## Introduction

### Community-Based Exercise Programs for People With Stroke

There are >13 million new cases of stroke per year worldwide [1], and 1 in 4 adults aged >25 years will experience a stroke in their lifetime [2]. Advances in acute stroke treatment have significantly reduced mortality; however, this increased survival rate has led to more people living with chronic stroke-related disabilities. With stroke now being a leading cause of long-term disability [3,4], rehabilitation researchers have identified enhancing brain recovery and promoting long-term healthy behaviors as a priority; this research has generated a wealth of new evidence-based stroke recovery practices [5,6]. However, there is a need to move this evidence into practice and close the gap between best and current stroke rehabilitation practices [7].

Although many individuals see improvements during the acute rehabilitation phase after the stroke, many lose their initial gains when they return to the community, and their disability progresses over time [8]. Evidence suggests that exercise improves motor function [9,10], health-related quality of life [11,12], cognitive function [13,14], and cardiovascular risk factors [15,16] in those with stroke. The implementation of community-based exercise programs, which are defined as “structured, instructional programs of exercise for groups or individuals delivered outside the public health care setting and available in community settings,” are avenues for engaging in lifelong physical activity [17]. Community programs that are focused primarily on walking, such as outdoor walking or mall walking programs, are often difficult to follow for individuals with mobility impairment from stroke, and many traditional fitness facilities and health clubs have accessibility barriers [18] that present additional challenges for people with stroke. Thus, people living with stroke may require adaptations to meet their unique needs and abilities. Stroke exercise programs should incorporate functional tasks that mimic daily activities, be guided by trained personnel knowledgeable in stroke and stroke-related impairments, and be delivered with an appropriate instructor-to-participant ratio [17]. Moreover, pre-exercise medical clearance by a health care provider and further eligibility screening by exercise providers are recommended to ensure the safety and appropriateness of adapted programs [17]. Despite the established benefits of ongoing exercise and evidence-based recommendations in the design and delivery of community-based exercise programs for stroke [17], most people with stroke do not have access to such community-based exercise programs that provide the specialized support to meet their long-term needs.

In 2016, the Heart and Stroke Foundation’s Canadian Partnership for Stroke Recovery (CPSR) [19] convened its Knowledge Translation Advisory Committee to identify priority areas for knowledge translation. The committee, comprising people with stroke, caregivers, stroke recovery experts, health care providers, policy makers, and knowledge translation and mobilization experts, identified poststroke exercise as a high priority and specifically identified the need to develop sustainable evidence-based and community-based exercise programs for people with stroke. Within Canada and internationally, researchers and clinicians have developed various community-based exercise programs for people with stroke [20-22]; there is now a need to catalyze the implementation of these evidence-based approaches to optimize the health and social benefits for people with stroke.

### Development of the Stroke Recovery in Motion Implementation Planner

**Figure 1:**
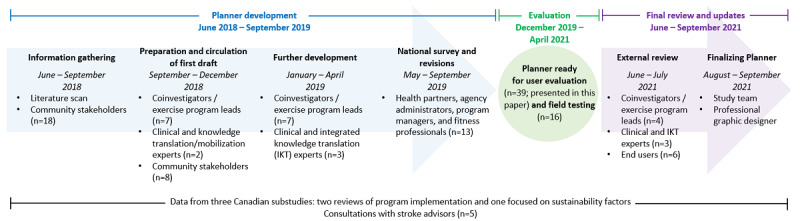
Summary of the Planner development process and stakeholders involved in the process.

Building on this momentum, our team aimed to develop an evidence-informed resource [23] to guide community program planners (eg, program managers and coordinators, rehabilitation health partners, fitness professionals, people with stroke, and caregivers) through the process of planning for the successful implementation of community-based exercise programs for people with stroke. We used a multistep process over 3 years to develop the Stroke Recovery in Motion Implementation Planner (hereafter referred to as the “Planner”; Figure 1).

The well-established Knowledge-to-Action (KTA) cycle, which helps bring the results of health care research into effective changes in practice, provided the overarching framework for the planning process [24-26]. It also builds on the CAN-IMPLEMENT guideline adaptation process, which divides the KTA cycle into 3 substantive planning phases [27,28]. Furthermore, the planning model incorporates elements of the Implementation Roadmap, which is based on the KTA cycle, and further breaks the 3 planning phases into practical steps and activities to facilitate implementation planning and execution [29]. The planning process is underpinned by 6 guiding principles (Textbox 1).

The “Planner development” phase (Figure 1) informed early decisions related to the content and organization of the Planner. For example, we identified the type of information and tools that would be most helpful to end users, decided on the use of a generic approach that could be applied to any stroke-specific community-based exercise program, worked to include real-world examples, and reduced technical language. As part of this development process, we conducted a national survey of potential end users across Canada (health partners, agency administrators, program managers, and fitness professionals). Of the 21 invited people, 13 (62%) reviewed the initial Planner prototype and completed a web-based questionnaire on what they liked and what could be improved.

As the prototype was developed, the research team engaged stroke advisors, including members from an existing group of Stroke Community Advisors through the CPSR. These stroke advisors (3 people with stroke and 2 caregivers of an individual with stroke) reviewed the Planner and provided feedback on the content and format to ensure that people with stroke, their families, and caregivers were reflected appropriately.

On completing this rigorous initial Planner development process, the resource was deemed *ready for evaluation (user evaluation and field testing)*.

**Implementation Planning Roadmap guiding principles underpinning the Stroke Recovery in Motion Implementation Planner.**

Characteristics of the planning approach:

- Intended for exercise programs *situated within the community* and provided by organizations with a mandate for community service (vs provision of individual therapeutic care)
- *Participant centered* (putting people with stroke and caregivers at the center of decisions and seeing them as experts working with service providers to achieve the best outcomes) [30]
- *Participatory and inclusive* (people with stroke and other relevant stakeholders, including health care partners, involved in cocreating the implementation plan)
- *Evidence-informed* (uses effective approaches to planning and implementation and incorporates the use of local data in making decisions)
- Aimed at *strengthening participant health outcomes*
- Focused on *sustaining successful programs*

### Study Objectives

The purpose of the study was to conduct a dual-component evaluation of the Planner comprising a user evaluation and field test. The objective of the user evaluation was to elicit user perceptions of the usefulness and acceptability of the Planner and revise the Planner based on the data. The objective of the field test was to describe how teams used the Planner in real-world conditions; describe the effects of using the Planner on participants’ implementation planning knowledge, attitudes, and activities; and identify factors influencing Planner use [31].

The goal of this paper (part 1) is to describe the results of the user evaluation and the revisions we subsequently made to the Planner. The results of the field test are reported separately in part 2 [31].

To guide the reporting of this study, we used guidelines for mixed methods studies (GRAMMS [Good Reporting of a Mixed Methods Study]) [32], qualitative studies (COREQ [Consolidated Criteria for Reporting Qualitative Studies]) [33], and survey studies (CROSS [Checklist for Reporting of Survey Studies]) [34]. These checklists are provided in Multimedia Appendix 1 [32-34].

## Methods

### Design

The user evaluation was a mixed methods study that used a concurrent triangulation design [35]. We used a convergent model where cross-sectional questionnaire data (closed and open-ended questions) and interview and focus group data were collected and analyzed separately, with the quantitative and qualitative findings merged during the interpretive phase [35]. The quantitative and qualitative data were assigned equal weight. The quantitative data facilitated identifying the “what”; specifically, it helped us identify what aspects of the Planner were most frequently identified as needing modification or removal, which enabled prioritization of essential changes. The qualitative data helped us understand the “why” of the participant feedback and provided insight into how we could best address participant concerns as we revised the Planner. The qualitative data also provided additional ideas to strengthen the Planner, which may not have been captured in the structured questionnaire. The settings for this study were Canada and Australia.

### Sampling and Recruitment

We used purposeful sampling. All staff involved or interested in the planning and delivery of community-based exercise programs for people with stroke were eligible to participate in the user evaluation, including community and municipal program directors, managers and coordinators, regional health authority staff, fitness or exercise professionals, physiotherapists, and other consulting health partners. We identified potential participants through the professional networks of (1) study coinvestigators, many of whom have developed exercise programs; (2) individuals who previously participated in the Planner development process; and (3) participants enrolled in the study (ie, snowball sampling).

In Canada, we aimed to identify participants from different geographical regions with various population densities. Using definitions from Statistics Canada [36], we created a matrix based on geographical region and population size category. As recruitment progressed, we aimed to identify individuals from the remaining undersampled cells (region × urban or rural) in our recruitment matrix. As the Canadian study progressed, we identified an emergent opportunity to include participants from Australia. One of the coinvestigators returned to Tasmania, Australia, where there was a state goal to increase community-based exercise opportunities for people with stroke. Unlike Canada, Tasmania had low rates of COVID-19 and fewer pandemic restrictions during the study period, and community-based program planning and implementation proceeded as usual. This gave us a unique opportunity to use the professional network of the coinvestigator, who was situated in an urban area of Tasmania, to identify participants who were currently actively engaged in the planning and delivery of community-based exercise programs for people with stroke. We consulted and received approval from our funding partner (CPSR) for the addition of participants from Australia, as there was perceived value in having an international perspective on the Planner.

We started recruiting individuals to prospectively field test the Planner in December 2019. In March 2020, the COVID-19 pandemic caused significant challenges for recruitment as the pandemic resulted in many teams stopping community-based program planning. In May 2020, we amended our protocol to add 2 additional groups (Table 1) to our study with different levels of experience in planning community-based exercise programs for people with stroke, allowing data collection on the Planner within pandemic restrictions. Study enrollment was completed in February 2021. The eligibility criteria for each participant group are presented in Table 1.

All 3 groups participated in the user evaluation component. The *current* program planners engaged in additional study activities as part of the field test component of the evaluation [31].

The study staff contacted potential participants via email with study information. If there was no response, we sent 2 follow-up reminders. Interested participants connected with the study staff to review the requirements, confirm eligibility, obtain informed written consent, and schedule a time for the study activities.

**Table 1:** Eligibility criteria and data collection methods for the 3 groups of participants in the study.

Participant group	Inclusion criteria	Data collection methods
*Current* program planners	Interested in implementing a community-based exercise program for people with stroke in the next 6 to 12 months Are willing to use the Planner to guide their planning process	Questionnaire Baseline interview or focus group Monitoring interviews End-of-study interview or focus group
*Future* program planners^a^	Have a vested interest in community-based exercise programs for people with stroke and the development of a useful resource for program planning Have not previously launched a community-based exercise program for people with stroke and are not currently considering planning a program	Questionnaire Follow-up interview or focus group
*Past* program planners^a^	Have previously implemented a community-based exercise program for people with stroke in the past 1 to 5 years	Interview or focus group about past experience Questionnaire Follow-up interview or focus group

^a^New participant group added in May 2020.

### Data Collection

The study participants completed a web-based questionnaire and a minimum of 1 interview. The order of the activities and the content of the questionnaire and interviews were tailored to each group (Table 1). Participants received an honorarium at a rate of CAD $25 (US $19.5) per hour to compensate for their time.

#### Questionnaire

Each participant completed a web-based questionnaire created in LimeSurvey (LimeSurvey GmbH) [37]. This was a “restricted” questionnaire, meaning that only participants enrolled in the study could complete the questionnaire through a single-use unique URL generated by the research team. There was no validated instrument that met our needs; therefore, the core study team created a questionnaire that was specific to the Planner content during the development phase (Figure 1). It was tested internally with 2 other research team members for functionality and clarity and then administered to 13 end users who represented people who were currently running programs, had run programs in the past, or were interested stakeholders. We then modified the questionnaire to tailor the content to the 3 participant groups and optimize functionality (eg, adding branching logic). Participants were required to read the Planner before starting the questionnaire. Depending on the group, the questionnaire had between 79 and 89 closed-ended questions, most of which also included open textboxes to expand on their selected answers, and between 11 and 22 open-ended questions. Questions were spread across 7 sections and focused on participants’ impressions of the Planner sections and tools (keep, modify, or remove), format and presentation, value of the Planner for community program planners, impact of the Planner on knowledge and confidence, and questions on respondents’ demographics and experience. The estimated time to read the Planner and complete the questionnaire was 4 hours. Multimedia Appendix 2 presents the questionnaires administered to the 3 study groups.

#### Interviews and Focus Groups

Each participant completed at least one interview or focus group. Some participants took part in the study as a team and therefore chose to complete a focus group together. The semistructured interview and focus group guides were developed by the research team and informed through discussions with end users in the Planner development phase. Question topics included overall impressions of the Planner, likes and dislikes regarding the Planner, in-depth discussion about the questionnaire responses, and perceived feasibility of the planning process. In addition, *past* program planners were asked to compare their current experience using the Planner with their previous experience. Multimedia Appendix 3 presents the semistructured interview and focus group guides.

*Current* program planners completed brief “monitoring” interviews that were scheduled every 1 to 2 months with the research staff. Discussion points during these interviews included how they had been using the Planner, what they liked about using it, and what was missing.

Interviews and focus groups were conducted either in person, through video calls, or by phone, depending on participant preference. Sessions were facilitated by 1 of 4 female researchers (1 master’s degree–prepared nurse researcher [J Reszel], 1 PhD-prepared rehabilitation researcher [TN], 1 master’s degree–prepared nutrition researcher [KE], and 1 PhD-prepared physiotherapy researcher [MLB]) experienced in qualitative data collection. None of them had pre-existing relationships with the participants. The interviewers worked to create a nonjudgmental environment and welcomed both positive and negative feedback. On average, the interviews and focus groups lasted 44 (range 21-106) minutes, and the monitoring interviews lasted 24 (range 13-39) minutes. The interviews were audio recorded and transcribed verbatim. Altogether, there were 42.9 hours of audio recordings, yielding 912 pages of transcripts. Field notes were made after the interviews and focus groups to document researcher reflections and observations, including participant interactions.

### Data Analysis

#### Questionnaire

We analyzed the data using descriptive statistics in SPSS (version 27; IBM Corp) [38]. For nominal data (eg, gender and geographical location) and ordinal data (eg, confidence and knowledge), we calculated frequencies and percentages to illustrate the distribution of the responses across categories. For continuous data (eg, years of experience), we calculated measures of central tendency (eg, mean and median) and measures of variability (eg, SD and range). The text in the open-ended questions was analyzed by grouping content into categories to identify what participants liked and disliked about the Planner steps, tools, format, and planning approach. Given the small sample size, no comparisons were made based on role, organization, or geography.

#### Interviews and Focus Groups

We used conventional content analysis, an inductive approach through which the codes and categories emerged from the data [39]. Each transcript was verified against the audio recording, read as a whole, and then segments of the text were labeled with codes in Microsoft Word. As the analysis progressed, we continued to develop our coding scheme while adding emerging codes. To enhance the trustworthiness of our findings, 40% of the transcripts were coded independently by 2 researchers [40]. The 2 researchers met regularly to compare their coding, resolve discrepancies, and update the coding scheme. All the transcripts were revisited to apply the final version of the coding scheme. We reached inductive thematic saturation [41] as no new codes were added after the 31st transcript (out of 67 transcripts). Data analysis occurred concurrently with data collection, with interview probes evolving to explore emerging themes from the analysis. The team reviewed and discussed field notes, and no notable group dynamics were identified. Although transcripts were not returned to participants, in the final stages of the study, 4 interview participants reviewed and shared their impressions of the revised Planner. This participant check allowed the study team to assess the extent to which we successfully applied the study findings to the Planner.

#### Triangulation of Quantitative and Qualitative Data to Inform Planner Revisions

On completion of the descriptive analysis of the questionnaire data and coding of the qualitative data, we created a master data summary document that included both data sets to facilitate comparing and contrasting. The core research team met regularly during this triangulation phase (>40 hours of meetings) and discussed the similarities and differences between the qualitative and quantitative data.

The team focused on changes to be made in the Planner, grounded in the study data. First, to identify “essential” changes, we reviewed the questionnaire data by using a threshold of 75%, a commonly used figure to define consensus [42]. Where <75% of respondents (in any of the 3 participant groups) selected the most positive response option for a question (ie, keep as is; strongly agree or agree), we carefully reviewed the open-ended questionnaire responses and qualitative interview data for that Planner section to determine the required changes. Next, even when consensus was reached (ie, ≥75%), we still reviewed every comment provided in the questionnaire and interviews to identify other opportunities to enhance the Planner. Decisions regarding whether to address the suggestions were based on (1) alignment with the guiding principles underpinning the Planner (Textbox 1), (2) alignment with evidence from implementation science (eg, best and promising practices for implementation) and pedagogical science (eg, value of repetition for learning), (3) the significance of the suggestions, and (4) the feasibility of the changes related to formatting and design constraints.

### Ethics Approval

We obtained ethics approval from the Ottawa Health Science Network Research Ethics Board (protocol 20190594-01H) and the Tasmania Health and Medical Human Research Ethics Committee (project ID 23559) for the initial and amended protocols. All participants signed an informed consent form before starting any study activities, and all study procedures were conducted in accordance with privacy and confidentiality requirements.

## Results

### Overview

We enrolled 39 participants between December 2019 and February 2021. We contacted 27 *current* program planners, of whom 16 (59%) enrolled; 14 *future* program planners, of whom 9 (64%) enrolled; and 43 *past* program planners, of whom 14 (33%) enrolled. The participant demographic data are presented in Table 2, participant roles based on employment setting are presented in Table 3, and the types of data collected during the study are presented in Table 4.

**Table 2:** Participant demographics (N=36)^a^.

Variable		Current program planners (n=15)	Future program planners (n=9)	Past program planners (n=12)	All participants
**Gender, n (%)^b^**					
	Female	4 (57)	8 (89)	10 (83)	22 (79)
	Male	3 (43)	1 (11)	2 (17)	6 (21)
	Gender fluid	0 (0)	0 (0)	0 (0)	0 (0)
**Location of community, n (%)**					
	Alberta	1 (7)	3 (33)	0 (0)	4 (11)
	British Columbia	4 (27)	0 (0)	2 (17)	6 (17)
	Manitoba	0 (0)	2 (22)	0 (0)	2 (6)
	Newfoundland and Labrador	1 (7)	0 (0)	0 (0)	1 (3)
	Nova Scotia	0 (0)	0 (0)	1 (8)	1 (3)
	Ontario	5 (33)	3 (33)	8 (67)	16 (44)
	Prince Edward Island	0 (0)	1 (11)	1 (8)	2 (6)
	Tasmania, Australia	4 (27)	0 (0)	0 (0)	4 (11)
**Population density, n (%)**					
	Rural or mostly rural	6 (40)	1 (11)	4 (33)	11 (31)
	Urban or mostly urban	7 (47)	7 (78)	5 (42)	19 (53)
	Combination of rural and urban	2 (13)	1 (11)	3 (25)	6 (17)
**Size of community, n (%)**					
	<5000	0 (0)	0 (0)	1 (8)	1 (3)
	5000-9999	5 (33)	0 (0)	0 (0)	5 (14)
	10,000-24,999	4 (27)	1 (11)	1 (8)	6 (17)
	25,000-50,000	1 (7)	0 (0)	0 (0)	1 (3)
	>50,000	5 (33)	8 (89)	10 (83)	23 (64)
**Type of organization where the program is or will be offered, n (%)^c^**					
	Community recreation center (public and municipal)	5 (33)	6 (67)	5 (42)	16 (42)
	YMCA^d^	1 (7)	0 (0)	5 (42)	6 (17)
	Community health center	1 (7)	2 (22)	1 (8)	4 (11)
	Recreation center for older adults	0 (0)	1 (11)	1 (8)	2 (6)
	Physiotherapy clinic	3 (20)	1 (11)	1 (8)	5 (14)
	Nursing home	0 (0)	0 (0)	1 (8)	1 (3)
	Retirement residence	0 (0)	0 (0)	2 (17)	2 (6)
	Private gym or facility	0 (0)	3 (11)	2 (17)	3 (8)
	Family health team	4 (27)	0 (0)	0 (0)	4 (11)
	Nonprofit community space	0 (0)	0 (0)	3 (25)	3 (8)
	Web-based program	2 (13)	1 (11)	1 (8)	4 (11)
**Number of years of experience in community program planning and/or delivery^b^**					
	Values, median (range)	2 (0-21)	5 (0-20)	10 (1-20)	7 (0-21)
	Values, mean (SD)	5 (8)	8 (6)	10 (6)	8 (7)
**Individual role in planning or delivering this exercise program, n (%)^e^**					
	Provider agency administration	0 (0)	1 (11)	1 (8)	2 (6)
	Program manager or coordinator	7 (47)	2 (22)	4 (33)	13 (36)
	Fitness or exercise professional	2 (13)	2 (22)	3 (25)	7 (19)
	Rehabilitation health professional	6 (40)	4 (44)	4 (33)	14 (39)
**Experience in *planning* this type of program, n (%)**					
	Previous experience planning adapted or specialized fitness programs	10 (67)	6 (67)	12 (100)	28 (78)
**Experience in *delivering* this type of program, n (%)**					
	Previous experience delivering adapted or specialized fitness programs	12 (80)	6 (67)	8 (67)	26 (72)
**Current confidence in planning an adapted or specialized fitness program, n (%)**					
	Extremely confident	0 (0)	1 (11)	1 (8)	2 (6)
	Very confident	5 (33)	4 (44)	6 (50)	15 (42)
	Moderately confident	8 (53)	3 (33)	5 (42)	16 (44)
	Slightly confident	2 (13)	1 (1)	0 (0)	3 (8)
	Not at all confident	0 (0)	0 (0)	0 (0)	0 (0)
**Knowledge of how to use evidence to inform decision-making in program planning, n (%)**					
	Extremely knowledgeable	3 (20)	0 (0)	0 (0)	3 (8)
	Very knowledgeable	4 (27)	8 (89)	3 (25)	15 (42)
	Moderately knowledgeable	2 (13)	1 (11)	8 (67)	11 (31)
	Slightly knowledgeable	4 (27)	0 (0)	1 (8)	5 (14)
	Not at all knowledgeable	2 (13)	0 (0)	0 (0)	2 (6)

^a^In this study, of the 39 participants, 36 (92%) completed the questionnaire. Of the 36 participants who completed the questionnaire, 35 (97%) questionnaires were complete, with only 1 (3%) participant skipping 2 out of 86 questions (the skipped questions are not reported in this table).

^b^The first version of the current program planner questionnaire did not include this question; therefore, 8 responses are missing.

^c^Respondents could select >1 response option.

^d^YMCA: Young Men's Christian Association.

^e^Some participants may actually represent >1 group (eg, a rehabilitation professional who is working as a program coordinator); however, these data reflect how participants self-identified their primary role in planning as per the questionnaire responses.

**Table 3:** Role of participants based on their employment setting (N=39).

Employment setting	Participant role, n (%)					Total, N
	Provider agency administration	Program manager or coordinator	Fitness or exercise professional	Rehabilitation health professional		
Community-based nonprofit^a^	2 (20)	4 (40)	4 (40)	0 (0)	10	
Municipality^b^	0 (0)	3 (60)	2 (40)	0 (0)	5	
Health authority^c^	0 (0)	2 (40)	0 (0)	3 (60)	5	
Private practice^d^	0 (0)	1 (17)	2 (33)	3 (50)	6	
Primary care^e^	0 (0)	1 (25)	0 (0)	3 (75)	4	
University	0 (0)	0 (0)	0 (0)	4 (100)	4	
Hospital	0 (0)	1 (50)	0 (0)	1 (50)	2	
Stroke network^f^	0 (0)	2 (67)	0 (0)	1 (33)	3	
Total	2 (5)	14 (36)	8 (21)	15 (38)	39	

^a^For example, the Young Men’s Christian Association (YMCA).

^b^For example, a city.

^c^For example, a provincial, state, or regional health authority.

^d^For example, a physiotherapy clinic or gym.

^e^For example, a family health team.

^f^For example, a provincial or regional network.

**Table 4:** Summary of quantitative and qualitative data collected by participant groups (N=39).

Data collection method	Current program planners (n=16^a^)	Future program planners (n=9^b^)	Past program planners (n=14^c^)	All participants
Questionnaire	15 responses	9 responses	12 responses	36 responses
Interviews and focus groups	15 interviews and focus groups with 16 participants	9 interviews with 9 participants	25 interviews and focus groups with 14 participants	49 interviews and focus groups with 39 participants
Monitoring interviews	18 interviews with 10 participants	N/A^d^	N/A	18 interviews with 10 participants

^a^From 7 planning teams.

^b^From 9 sites.

^c^From 13 sites.

^d^N/A: not applicable.

### Overall Impressions of the Planner

Overall, feedback on the Planner was positive. All questionnaire participants felt that the Planner addressed the key factors to consider when planning a community-based exercise program for people with stroke; almost all (34/36, 94%) felt it would help them make decisions informed by evidence, and most (29/36, 81%) indicated that it would improve their usual approach to planning:

The Planner is a very good teaching tool and I think you could easily work through that with a team. It would put your team all on the same page and certainly enhance some people’s background...I think it would keep the group on the same page and have a good idea of what we’re doing, why we’re doing it, who we’re doing it for, and the benefits.

Fitness or exercise professional, ID38

Most participants (26/28, 93%) reported that reading the Planner increased their knowledge of how to use evidence to inform decision-making in program planning. Nearly as many participants (24/28, 86%) also reported that reading the Planner increased their confidence in their ability to plan an adapted or specialized fitness program.

Several *past* program planners who had successfully launched and sustained programs commented on the alignment between the Planner and their own experiences and the added benefit of the Planner:

Initially it [the Planner] was overwhelming and I went “oh my gosh I missed every single step”...But then I looked back and I saw that I did most of it, but just not necessarily with as much intention or thought. A lot of it [Planner content] is just kind of intuitive and I did it but it wasn’t as thoughtful.

Rehabilitation health professional, ID37

Nearly all *future* and *past* program planners (19/21, 91%) indicated that they would likely use the Planner for future program planning. Similarly, most questionnaire participants (33/36, 92%) said they would likely recommend it to colleagues to support program planning, described by one participant as follows:

If I knew somebody was thinking of [planning a program], I think a resource such as this would be the exact one they should be using. If I were in a position where I was implementing or supporting or advising on the implementation of a program, I would definitely recommend the use of this resource at the planning stage in order to make sure that everything has been thought about.

Rehabilitation health professional, ID21

Most questionnaire participants (34/36, 94%) agreed that the Planner could be applied to planning programs that were not stroke specific, including exercise programs for people with other health conditions and nonexercise programs:

I’m working on a different program and some of the concepts in the Planner have helped develop that program. It’s not an exercise program but the Planner has helped me think about other things when it comes to programming.

Program manager or coordinator, ID12

Although the most commonly reported first impression of the Planner was that it was long and possibly overwhelming, over three-quarters (28/36, 78%) of the questionnaire participants agreed that the Planner presented the right amount of information. Most participants indicated that after reading and digesting the information, they saw great value in all the presented materials and could not identify materials that could be removed. Participants recognized the need to strike a balance between making the Planner a comprehensive information source for people with various roles and experience levels while also ensuring that the material was not overly long or onerous to read:

As far as usability, it’s trying to walk that fine line between providing too much information and not enough information. Because you get a variety of people, from those who have never implemented a community-based program, to those who are very used to that. Trying to make it work for both those groups, how do you do that as best as possible? That’s not an easy answer.

Program manager or coordinator, ID13

All questionnaire participants (36/36, 100%) agreed that the Planner was well organized, and nearly all agreed that it was easy to read and understand (35/36, 97%) and clearly presented the planning process (33/36, 92%). Participants were split on the format of the Planner: 56% (20/36) of participants would have preferred a web-based version over a paper-based version, and 28% (10/36) did not have a preference. Participants frequently desired access to both a web-based version for easy navigation and tool completion and a paper-based version for hard copy use.

### Feedback That Prompted Changes to the Planner

#### Planner Content

There were several recurring comments related to the Planner content, which cut across sections and tools that resulted in edits. For example, we observed a discordance in some participant settings between the Implementation Planning Roadmap and the “usual way things are done,” reflecting a difference in philosophy. To address this, we made the guiding principles and assumptions explicit at the beginning of the Planner to make the approach and values transparent and explain the rationale for the planning process (Textbox 1). Furthermore, some participants expressed concerns about the theoretical language used in the Planner. We carefully reviewed the Planner and simplified the technical terms wherever possible.

For questions assessing specific Planner sections and tools, 70% (30/43) of the items were deemed “necessary—keep as is” by at least 75% of the questionnaire participants (Multimedia Appendix 4). Key content changes were made to the Planner and tools based on the participant feedback (Table 5).

**Table 5:** Examples of key content changes made to the Planner and tools.

Identified area of improvement	Changes made to the Planner and tools
Include more information to clarify why specific steps and activities are important to complete during implementation planning (eg, forming planning partnership, decision-making methods, terms of reference, celebrating the launch, and preparing an evaluation plan)	“Why is this important” statements were emphasized throughout the Planner to provide the rationale and potential benefits of completing the step or activity
Include more examples of the real-world solutions used by other teams to address planning challenges; include examples of completed tools from planning teams	Addition of the “Tips and Potholes” section at the end of each planning phase to highlight the success factors and challenges encountered by teams involved in the development and evaluation of the Planner Added samples of completed tools created by study sites (with permission)
Wherever possible, make content action oriented	Implementation Planning Roadmap revised from 13 steps to 8 steps and Planner guidance edited to provide greater clarity and focus on specific activities and tasks to complete All tools reviewed and edited to ensure templates provide concrete guidance Creation of standardized cover sheets for each tool, which include “Why is this important?” and “How to use this tool” statements
Include information on how to consider the specific needs of people with stroke or caregivers as planning partners	New section and tool with specific guidance on factors to consider and questions to ask when engaging people with stroke and caregiver partners in the team Voices of people with stroke and caregivers were brought to the forefront by inserting verbatim quotes collected during our evaluation throughout the Planner
Include more exercise program–specific information to facilitate program comparisons	Creation of a “program comparison template” with guiding questions for planning teams to assess the history, attributes, and requirements of programs under consideration
Emphasize the importance of considering and addressing program sustainability factors early and often	Sustainability information was included in all 3 phases of the Planner Creation of a new section on sustainability capacity Key sustainability factors identified in the end-of-phase checklists and throughout tools
Make tools concise (eg, implementation work plan and assessment of barriers) and avoid duplication between tools (eg, community assessments)	Tool content reorganized, simplified, and relabeled to align more clearly with road map steps Repetitive content merged and the number of tools reduced Longer tools split into easy-to-manage sections (eg, identifying barriers to program, program users, and program setting became 3 short worksheets)

#### Planner Format and Organization

The participants offered constructive comments on how to optimize the format and organization of the Planner, which resulted in several key changes (Table 6).

**Table 6:** Examples of key format and organization changes made to the Planner and tools.

Identified area of improvement	Changes made to the Planner and tools
Simplify structure, balance the workload across the 3 phases, and reorder the sequence of activities and steps	Implementation Planning Roadmap reduced and simplified from 13 steps to 8 steps Implementation planning process reorganized to better balance planning activities within and across the 3 phases Phase 2 and 3 steps reordered to make the planning sequence more logical (eg, developing an evaluation plan before launching the exercise program)
Improve navigation; clearly align Planner content with the phases and steps of the road map	Road map figure moved to the start of the Planner as a key navigation element Planner redesigned to better link content to road map phase and step and orient the reader to the location on the map Professional graphic design concept developed to facilitate navigation
Facilitate different “starting points” in the Planner to help situate readers from different contexts and starting places in their planning journey	Developed a new “Where do we start?” section in the Planner introduction to outline different planning scenarios and potential starting points and how to use the Planner accordingly Directed readers to the progress checklists at the end of each phase to assess what work still needs to be completed
Keep the body of the Planner concise for easy reading	Selected content (eg, additional resources and program samples) moved from the body of the Planner to the appendix as “Read more” sections for interested readers
Provide easy access to tools and appendices (additional resources); ensure tools are fillable and editable	Tools summarized at end of each phase with links PDF and original, editable files provided for easy download Design concept to include both hard copy and web-based versions of the Planner

### Feedback Considered but Existing Approach Maintained

The study participants made some suggestions for Planner modifications that after careful consideration, we decided not to make. Here, we provide 2 key examples with the rationale.

First, some participants requested that to facilitate use, the Planner should be separated into different sections to assign specific content to different roles on the planning team. However, the desire to distinguish between those “planning” and those “delivering” was not aligned with our guiding principle of using a participatory and inclusive approach. Planning teams are most effective when those delivering the program (eg, fitness or exercise professionals) and using the program (eg, people with stroke) are engaged early in the planning process. In addition, team members’ roles may be fluid over time and in different contexts. Therefore, we left the Planner as one document designed for all team members, with the goal of encouraging awareness of and participation in the full planning process. The importance of an integrated planning team was supported by a study participant who regularly delivered community programming:

I was really excited about having “boots on the ground” kind of people [on the planning team]. Because often times you don’t hear about these things until after others have made the decision and you’re like “oh if only you had talked to the people who actually implement these things. It would have been helpful.” Because the concept of policy can often vary greatly from the reality.

Fitness or exercise professional, ID22

Second, several participants commented on the level of repetition throughout the Planner and recommended making the document shorter and more concise. Although we carefully reviewed the Planner and eliminated unnecessary redundancies, some repetition was left in for pedagogical reasons [43]. Repetition helps readers remember and understand the information. We also recognized that some readers would not read the Planner from start to finish and skip from section to section; therefore, we chose to judiciously repeat essential contextual information throughout the Planner.

### Positive Feedback on the Planner Supporting the Existing Approach and Resulting in Enhancements

All the participants provided positive feedback on the Planner and identified features that they found useful. We used this positive feedback to identify content and features to keep in the final version. We also identified ways of expanding upon and further enhancing these features where possible (Table 7).

**Table 7:** Sections and features of the Planner rated positively with illustrative quotes.

Positive feedback on the Planner	Illustrative quote	Planner decisions made based on positive feedback
Although many participants felt they would not necessarily need to use all Planner tools to implement every exercise program, they generally appreciated the inclusion of various tools, should they be needed.	“Although I feel all [tools] are important to keep, I don’t feel I would use them all each time I would start a program. It would depend on the type of organization I was working with and how much detail would be needed, thus having all the tools available is important.” [Rehabilitation health professional, ID32]	Kept a variety of tools to meet the needs and contexts of different planning teams Created cover pages for each tool, further highlighting who, how, when, and why planning teams can use the various tools
Although many participants felt the Planner was long, most participants appreciated the comprehensiveness of the Planner and the breadth of information presented.	“There’s lots of information. You can go lots of places to look at program planning information, but having it all consolidated...is really helpful to me. Because I could get lost and I could go down a significant rabbit hole if I start Googling all this stuff on my own. To forego the Google rabbit hole is very helpful.” [Program manager or coordinator, ID35]	Kept the Planner as a comprehensive document to meet the needs of various planning team members New content added based on participant feedback to improve comprehensiveness; for example, more details on developing a planning partnership, how to engage people with stroke and caregiver partners, and web-based program information
Nearly all participants commented positively on the summary checklists at the end of each phase as a clear way of assessing progress and the remaining planning tasks.	“I did like the progress checklists. I really liked that at the end of each section. It was a nice way to kind of bring all of that together and in a practical tool that people can use.” [Rehabilitation health professional, ID33]	Kept checklists at the end of each phase Phase checklists were made into a separate tool for easy access and printing Content of checklists was integrated into the implementation work plan
Many participants valued the quotes and field notes from other planning teams to learn about real-world successes and challenges and highlight the importance of the various planning steps.	“I liked the field notes about programs—this is what happened and this is the result...It makes it relatable; when you’re reading all the info, it pulls you back into the practical side of it, which is good.” [Fitness or exercise professional, ID31]	New quotes from study participants added throughout the Planner “Tips and Potholes” added to the end of each phase to further highlight study participant experiences and learnings

### Planner Revisions

**Figure 2:**
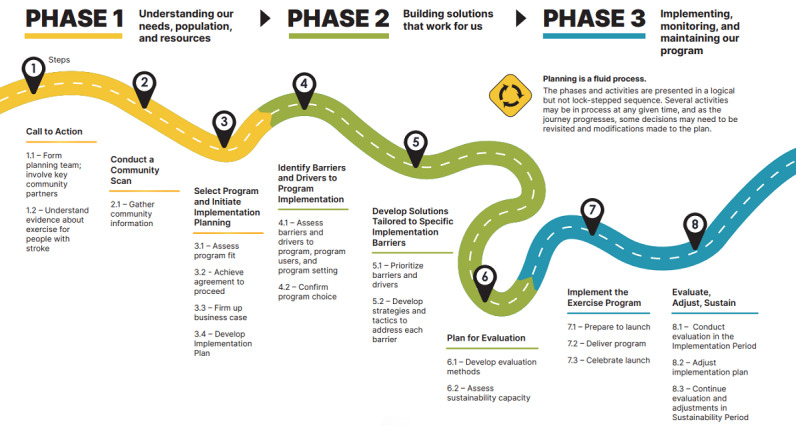
Implementation Planning Roadmap from the Planner summarizing the phases, steps, and activities.

Using the data reported in this study, we revised both the content and format of the Planner. Integrating this feedback involved extensive rewriting and editing by the core study team; contracting the graphic designer; and a final review by 16 coinvestigators, consultants, end users, and stroke advisors. The largest revision of the Planner involved improving the overall structure and navigation (Figure 2).

The Stroke Recovery in Motion Implementation Planner is now hosted through March of Dimes Canada, a Canadian nonprofit organization offering services for people with disabilities, including the After Stroke program focused on stroke recovery in the community [44]. The Planner is free and publicly available here [23].

## Discussion

### Principal Findings

This paper provides a comprehensive description of how the Planner was developed, informing consumers of the rigorous process used. Our mixed methods user evaluation demonstrated that, overall, end users viewed the Planner positively and indicated that it was a useful and valuable resource. They reported that it would improve the planning process and help them make planning decisions informed by evidence. The participants’ constructive feedback on the content and organization was used to revise and strengthen the Planner.

### Comparison With Prior Work

Our development process aligns with that used by others to develop implementation guides and toolkits in different settings, including selecting an underpinning theory or framework [45-48], searching the academic and gray literature [46-50], consulting experts and consumers [45-48,50], and refining the guide based on end user feedback [45,46,48-50].

Some participants provided feedback consistent with findings reported in the literature; for example, perceptions that the implementation process could be overwhelming or the guide too long [50,51], the need to reduce the number of steps and technical language [51], and the desire for new content and tools to address team needs [48,49,51]. Engaging a wide variety of stakeholders in the development process and using several user-centered design strategies ensured that the final product was grounded in the needs and experiences of those who will use it in real-world settings [52].

The use of an implementation framework can contribute to more systematic planning, delivery, and evaluation of programs, thereby contributing to improved success and sustainability [53]. However, many implementers lack knowledge and experience in using these frameworks [53], and many third-sector organizations (eg, voluntary and community organizations and social enterprises [54]) face capacity and capability issues when implementing evidence-based interventions [55]. Leeman et al [56] identified “tools” as a strategy for building implementation capacity in community-based practitioners. The Planner is an evidence-informed tool for building the capacity of practitioners (in this context, community program planners, health professionals, fitness professionals, people with stroke, and caregivers) to plan for implementation in an applied and approachable way. Most study participants reported an increase in knowledge and confidence after reading the Planner, including many who reported being experienced program planners.

The Planner is based on the KTA, CAN-IMPLEMENT, and Implementation Roadmap frameworks [24,25,27-29] and is grounded in implementation science and practice. Studies on third-sector organizations in general [55] and on poststroke exercise specifically [57-60] have revealed factors that can influence program implementation, such as equipment, space, time, staffing, training, funding, marketing and recruitment, class capacity, sustainability, program adaptation challenges, organizational culture and priorities, and collaboration between organizations and professionals [55,57-60]. The Planner is specifically designed to help planning teams identify these and other factors unique to their settings, which may impede or support the implementation of community-based exercise programs for people with stroke. The Planner offers strategies, including some recommended by others [55,57], to overcome these barriers. Most importantly, the Planner provides a step-by-step action-oriented road map to plan for successful implementation and sustainability.

### Limitations and Strengths

This study has some limitations that should be acknowledged. A key challenge was conducting the study during the COVID-19 pandemic. Although we successfully recruited 39 participants to review the Planner, the data collection period coincided with the pandemic, a time of high personal and professional stress. It is unknown how the stress of the pandemic may have influenced decisions to participate in the evaluation or how it influenced participants’ perspectives of the Planner. However, nearly half of the individuals approached to participate did so despite the burden of having to read the comprehensive Planner, complete a lengthy questionnaire, and participate in an interview. This suggests that those who participated were strongly committed to providing input on the Planner and provided thoughtful and detailed feedback.

Although our sample was diverse, we would have liked to have enrolled more fitness professionals, given their role in delivering exercise programs. There may be several reasons for their limited enrollment. For the *current* program planning group, our primary contact was often the manager or coordinator or health partner. These planning leads had sometimes not yet identified the fitness professional team members and were unable to connect the research team with fitness professional study candidates. Furthermore, the pandemic led to the closure of community centers and fitness facilities, and therefore, many fitness professionals were not actively employed during this time. Despite these challenges, approximately 20% (8/39) of the study participants were fitness or exercise professionals who offered rich and thoughtful guidance on how to improve the Planner to meet their needs.

Finally, the user evaluation involved the hard copy version of the Planner, and therefore, the findings may not reflect perceptions of a web-based format. Plans are underway to develop a web-based toolkit based on the hard copy version.

A strength of the study was the mixed methods design, which facilitated assessing perceptions of the Planner through a comprehensive questionnaire with standard questions for all participants, followed by an interview for in-depth discussions on dimensions that were particularly important to each participant. We used multiple types of triangulation, including methods (questionnaires and interviews), sources (participants from diverse settings), and analysts (coding and interpretation by multiple researchers), all of which enhanced the quality and credibility of our findings [61]. The targeted enrollment of stakeholders from 3 groups (*current*, *future*, and *past*) allowed us to collect data (and reach saturation) from a broad range of stakeholders in various geographical areas with differing experiences in community-based exercise programs for people with stroke. Our rigorous analysis process resulted in every comment being reviewed and carefully considered to inform the Planner revisions and facilitated improved relevance and feasibility of the Planner.

### Future Directions

With the Planner finalized and freely and publicly available for use by community teams, we now have the opportunity to further evaluate its ongoing use and impact. Access and use will be monitored through website statistics and consumer inquiries. Working with key stakeholders, we also plan to augment the Planner to better address culturally tailored physical activity programs for racialized populations with stroke, as well as issues related to planning web-based programs for people with stroke. Finally, future work will involve developing products that distill the Planner information into alternate formats to meet diverse learning needs, including video vignettes, infographics, a condensed pocket guide, and a presentation slide deck.

### Conclusions

Community-based exercise programs are urgently required to address community reintegration and transitions for people living with the effects of stroke. The Stroke Recovery in Motion Implementation Planner [23] was designed to address the limited use of evidence-informed planning practices for community-based exercise programs for people with stroke. Guided by knowledge from the field of implementation science on how to facilitate implementation, we used a rigorous process to develop and evaluate the Planner. The evaluation revealed that the Planner was perceived to be a valuable resource that may be used to guide interdisciplinary teams in the planning and delivery of evidence-informed, sustainable, community-based exercise programs for people with stroke.

## Appendix

Multimedia Appendix 1 Reporting checklists.

Multimedia Appendix 2 Questionnaires for current, future, and past program planners.

Multimedia Appendix 3 Semistructured interview and focus group guides.

Multimedia Appendix 4 Questionnaire data on Planner sections and tools according to the study group.

## Abbreviations

COREQ: Consolidated Criteria for Reporting Qualitative Studies
CPSR: Canadian Partnership for Stroke Recovery
CROSS: Checklist for Reporting of Survey Studies
GRAMMS: Good Reporting of a Mixed Methods Study
KTA: Knowledge-to-Action
