# Supplementary material for: The Stroke Recovery in Motion Implementation Planner: Mixed Methods User Evaluation
Source: JMIR Form Res. 2022 Jul 29;6(7):e37189. doi: 10.2196/37189 (PMC9377478; doi:10.2196/37189)
Supplement: Multimedia Appendix 2 [file formative_v6i7e37189_app2.pdf]

Multimedia Appendix - Questionnaires for *Current, Future,* and *Past* Program Planners

This is a Multimedia Appendix to a full manuscript published in the JMIR Form Res. For full copyright and citation information see http://dx.doi.org/10.2196/37189

CURRENT Program Planner Questionnaire
PART A: CONTENT OF GUIDE

Based on your review of the planning material and the content associated with each of the planning elements listed below, select **one statement** which best describes your impression. Please provide comments or suggestions for modifications where you think it is necessary. There is space for your input at the end of each section.

Guide Phase 1: Understanding our needs, population, and resources

1)	Please select one statement per row		
	**Necessary: keep, as is**	**Necessary: needs modification** *(please explain below)*	**Not necessary: remove** *(please explain below)*
A1 - Explore the "call to action"; thinking about starting a program (p. 2-3)	○	○	○
A2 - Involve the community; identify key partners (p. 3-5)	○	○	○
A2 - Develop your teams' terms of reference; creating a project charter (p. 6)	○	○	○
A3 - Form consensus about the importance of exercise for people with stroke (p. 7-8)	○	○	○
A3 - Features of exercise programs designed for people with stroke (p. 8-11)	○	○	○
A4 - Conduct community assessment (p. 12)	○	○	○
A5 - Choose a program that is a good fit for your community (p. 13-14)	○	○	○
A5 - Cost implications (p. 14-16)	○	○	○
Fig. 3 - Decision making: should we and can we proceed with an	○	○	○

1)	Please select one statement per row		
	**Necessary: keep, as is**	**Necessary: needs modification** *(please explain below)*	**Not necessary: remove** *(please explain below)*
exercise program here? (p. 17)			
A6 - Prepare your business case (p. 18)	○	○	○
A6 - Prepare your implementation work plan (p. 19)	○	○	○
A7 - Achieve agreement to proceed (or not) (p. 19)	○	○	○

1a) If you indicated that any of the Phase 1 sections "need modification" or should be "removed", please explain below.

1b) Please provide any additional comments or suggestions for the Phase 1 guide content.

Guide Phase 2: Building solutions that work for us

2)	Please select one statement per row		
	**Necessary: keep, as is**	**Necessary: needs modification** *(please explain below)*	**Not necessary: remove** *(please explain below)*
B - Assess barriers and drivers to program implementation (p. 22-28)	○	○	○
C - Address identified challenges to program implementation (p. 28-34)	○	○	○

2a) If you indicated above that any of the Phase 2 sections "need modification" or should be "removed", please explain below.

2b) Please provide any additional comments or suggestions for the Phase 2 guide content.

Guide Phase 3: Implementing, monitoring, and maintaining our program

3)	Please select one statement per row		
	**Necessary: keep, as is**	**Necessary: needs modification** *(please explain below)*	**Not necessary: remove** *(please explain below)*
D - Prepare to launch the program (p. 37-38)	○	○	○
D - Celebrate the launch (p. 38)	○	○	○
E - Develop an evaluation plan; monitoring program delivery and use (p. 39-41)	○	○	○
F - Assess participant and program outcomes (p. 42-46)	○	○	○
G - Ensure continued operation of the program and respond to change (p. 46-48)	○	○	○

3a) If you indicated above that any of the Phase 3 sections “need modification” or should be “removed”, please explain below.

3b) Please provide any additional comments or suggestions for the Phase 3 guide content.

PART B: INDIVIDUAL TOOLS AND RESOURCES

For each of the tools and resources listed below, select **one statement** which best describes your impression. Please provide comments or suggestions for modifications where you think it is necessary. There is space for your input at the end of the section.

Stroke Recovery in Motion Planner Tools and Resources

4)	**Please select one statement per row**		
	**Necessary: keep, as is**	**Necessary: needs modification** *(please explain below)*	**Not necessary: remove** *(please explain below)*
Implementation Planning "Roadmap" (Phase 123 diagram, p. 53)	○	○	○
Project Charter: Template (p. 54)	○	○	○
Declaration of Conflict of Interest: Sample Disclosure (p. 55-57)	○	○	○
Community (Environmental) Scan: Worksheet (p. 58)	○	○	○
Community/Environmental Readiness: Worksheet (p. 59)	○	○	○
Sample Questions to Assess Community Readiness (p. 60-62)	○	○	○
Feasibility, Applicability, Acceptability: Checklist (p. 63-65)	○	○	○
Budget Planning Worksheet: Sample (p. 66-68)	○	○	○
Preparing the Business Case: Template (p. 69-70)	○	○	○
Implementation Work plan: Template (p. 71-73)	○	○	○

4)	Please select one statement per row		
	**Necessary: keep, as is**	**Necessary: needs modification** *(please explain below)*	**Not necessary: remove** *(please explain below)*
Identification of Barriers and Drivers: Template for Solution Building (p. 74-77)	○	○	○
Medical Authorization: Fit for Function Program Sample (p. 78-79)	○	○	○
Recreation Centre Readiness Checklist: TIME Program Sample (p.80-81)	○	○	○
Participant Fitness Progress Log: Fit for Function excerpt (p. 82-83)	○	○	○
Program Sustainability Assessment Tool (p. 84-87)	○	○	○
Glossary (p. 88-91)	○	○	○
Appendix: The Knowledge to Action Cycle (p. 92-93)	○	○	○
Appendix: Decision-Making (p. 94-96)	○	○	○
Appendix: FAME, Fit for Function, TIME and HWE exercise program information (p.97-103)	○	○	○
Appendix: Defining Indicators (p.104)	○	○	○
Appendix: Outcome Measures (p. 105-107)	○	○	○
Appendix: Short Physical Performance Battery from FAME exercise program (p. 108)	○	○	○
Appendix: Sample Program Fidelity Checklists from FAME, Fit for Function, and TIME programs (p. 109-114)	○	○	○
Bibliography (p. 115)	○	○	○

4a) If you indicated above that any of the tools or resources in the Planner "need modification" or should be "removed", please explain below.

4b) Please provide any additional comments or suggestions for the tools or resources in the Planner.

PART C: FORMAT AND PRESENTATION

Please **select one statement** (on the scale between Strongly Agree to Strongly Disagree) to rate your impression of the usability of the Planner.

The Stroke Recovery in Motion planning material...

5)	Please select one statement per row				
	**Strongly Agree**	**Agree**	**Neither Agree or Disagree**	**Disagree**	**Strongly Disagree**
is well organized	○	○	○	○	○
is written in plain language, easy to read and understand	○	○	○	○	○
clearly presents the planning process	○	○	○	○	○
clearly presents the research evidence for exercise	○	○	○	○	○
provides realistic, useful planning examples, tips, and stakeholder perspectives	○	○	○	○	○
includes helpful illustrations, pictures	○	○	○	○	○
the Phase 1-2-3 'Progress Checks' are helpful	○	○	○	○	○
provides easy access to worksheets, tools, templates and added resources	○	○	○	○	○
is user-friendly and easy to navigate	○	○	○	○	○
presents the right amount of information	○	○	○	○	○
I would prefer to use an on-line, web-based, interactive version vs. a paper version of the Planner	○	○	○	○	○

5a) Comments:

PART D: VALUE OF THIS RESOURCE FOR ASSISTING COMMUNITY PROGRAM PLANNERS

The Stroke Recovery in Motion Planner...

6)	Please select one statement per row				
	**Strongly Agree**	**Agree**	**Neither Agree or Disagree**	**Disagree**	**Strongly Disagree**
addresses the key factors to consider when planning an exercise program for people with stroke	○	○	○	○	○
uses a planning model supported by research	○	○	○	○	○
will help us make decisions informed by evidence	○	○	○	○	○
conflicts with our usual approach to planning	○	○	○	○	○
is compatible with our usual approach to planning	○	○	○	○	○
will improve our usual approach to planning	○	○	○	○	○
will require changes to our usual approach to planning	○	○	○	○	○
is suitable for most program planning scenarios	○	○	○	○	○
could be applied to planning for other programs	○	○	○	○	○
will meet the needs of community planners	○	○	○	○	○
will improve the sustainability of our program	○	○	○	○	○
will improve program outcomes	○	○	○	○	○

6a) Comments:

PART E: GENERAL IMPRESSIONS

Comment on the following (please write on reverse if you need more space):

7) What I like most about the planning approach and/or materials:

8) What I like least about the planning approach and/or materials:

9) What is missing from the Planner:

10) My recommendations for improving the Planner:

11)	Please select one statement per row				
	Very likely	Likely	Neutral	Unlikely	Very unlikely
How likely is it that you would provide the Planner to other colleagues to support program planning?	○	○	○	○	○

PART F: YOUR COMMUNITY AND EXERCISE PROGRAM PLANS

Please tell us about your community, your role / experience, and current plans for introducing a community-based exercise program for people with stroke.

12) Location of community:

Please choose **one** of the following answers:

- ○ Alberta
- ○ British Columbia
- ○ Manitoba
- ○ New Brunswick
- ○ Newfoundland and Labrador
- ○ North West Territories
- ○ Nova Scotia
- ○ Nunavut
- ○ Ontario
- ○ Prince Edward Island
- ○ Quebec
- ○ Saskatchewan
- ○ Yukon Territory
- ○ Other (please specify):

13) Population density:

Please choose **one** of the following answers:

- ○ Rural or mostly rural
- ○ Urban or mostly urban
- ○ Other - please describe:

14) Please enter the first three characters of your organization's postal code:_______________

15) Size of community

Please choose one of the following answers:

- ○ less than 5,000
- ○ 5,000 - 9,999
- ○ 10,000 - 24,999
- ○ 25,000 - 50,000
- ○ more than 50,000

16) Indicate the type of organization / facility in which you anticipate offering the exercise program:

Check all that apply:

- ☐ Community Recreation Centre (e.g. Public, municipal)
- ☐ YMCA
- ☐ Community health centre
- ☐ Seniors recreation centre
- ☐ Physiotherapy clinic
- ☐ Nursing home
- ☐ Retirement residence
- ☐ Private gym
- ☐ Other - please describe:

PART G: DEMOGRAPHICS & YOUR EXPERIENCE

The Planner was designed to help users understand evidence-based decision-making for program planning and the requirements for adapted and specialized programs. We are interested in learning about your experience with this approach to planning.

17) What is your gender?

Please choose one of the following answers:

- ○ Male
- ○ Female
- ○ Gender fluid
- ○ Other
- ○ Prefer not to answer

18) How many years of experience do you have in community program planning and/or delivery?

______________________ years

19) What is your individual role in planning or delivering this exercise program?

Please choose one of the following answers:

- ○ Provider Agency Administration
- ○ Program Manager/Coordinator
- ○ Fitness Instructor
- ○ Health Partner
- ○ Sponsoring Partner
- ○ Participant
- ○ Other - please describe:

20) What is your experience in *planning* this type of program?

Please choose one of the following answers:

- ○ First time
- ○ Previous experience planning adapted or specialized fitness programs

Please comment on your choice here if needed:

21) What is your experience in *delivering* this type of program?

Please choose one of the following answers:

- ○ First time
- ○ Previous experience planning adapted or specialized fitness programs
- ○ Not applicable

Please comment on your choice here if needed:

22) Please rate your current confidence in *planning* an adapted or specialized fitness program:

Please choose one of the following answers:

- ○ Not at all confident
- ○ Slightly confident
- ○ Moderately confident
- ○ Very confident
- ○ Extremely confident

23) Has reading the Planner had any effect on your confidence in your ability to plan an adapted or specialized fitness program?

Choose one of the following answers:

- ○ Yes, reading the Planner *increased* my confidence
- ○ Yes, reading the Planner *decreased* my confidence
- ○ No, reading the Planner had *no effect* on my confidence

24) How knowledgeable are you of how to use evidence to inform decision-making in program planning?

Please choose one of the following answers:

- ○ Not at all knowledgeable
- ○ Slightly knowledgeable
- ○ Moderately knowledgeable
- ○ Very knowledgeable
- ○ Extremely knowledgeable

25) Has reading the Planner improved your knowledge of how to use evidence to inform decision-making in program planning?

Choose one of the following answers:

- ○ Yes, reading the Planner *increased* my knowledge
- ○ No, reading the Planner had *no effect* on my knowledge

26) Is there anything else you would like to tell us?

Thank you for your input!

FUTURE Program Planner Questionnaire
PART A: CONTENT OF GUIDE

Based on your review of the planning material and the content associated with each of the planning elements listed below, **select one statement** which best describes your impression. Please provide comments or suggestions for modifications where you think it is necessary. There is space for your input at the end of each section.

Guide Phase 1: Understanding our needs, population, and resources

1)

	Please select one statement per row		
	**Necessary: keep, as is**	**Necessary: needs modification** *(please explain below)*	**Not necessary: remove** *(please explain below)*
A1 - Explore the "call to action"; thinking about starting a program (p. 2-3)	○	○	○
A2 - Involve the community; identify key partners (p. 3-5)	○	○	○
A2 - Develop your team's terms of reference; creating a project charter (p. 6)	○	○	○
A3 - Form consensus about the importance of exercise for people with stroke (p. 7-8)	○	○	○
A3 - Features of exercise programs designed for people with stroke (p. 8-11)	○	○	○
A4 - Conduct community assessment (p. 12)	○	○	○
A5 - Choose a program that is a good fit for your community (p. 13-14)	○	○	○
A5 - Cost implications (p. 14-16)	○	○	○
Figure 3 - Decision making: should we and can we proceed with an exercise program here? (p. 17)	○	○	○
A6 - Prepare your business case (p. 18)	○	○	○

	Please select one statement per row		
	**Necessary: keep, as is**	**Necessary: needs modification** *(please explain below)*	**Not necessary: remove** *(please explain below)*
A6 - Prepare your implementation work plan (p. 19)	○	○	○
A7 - Achieve agreement to proceed (or not) (p. 19)	○	○	○

1a) If you indicated above that any of the Phase 1 sections "need modification" or should be "removed", please explain below.

1b) Please provide any additional comments or suggestions for the Phase 1 guide content.

Guide Phase 2: Building solutions that work for us

2)

	**Please select one statement per row**		
	**Necessary: keep, as is**	**Necessary: needs modification** *(please explain below)*	**Not necessary: remove** *(please explain below)*
B - Assess barriers and drivers to program implementation (p. 22-28)	○	○	○
C - Address identified challenges to program implementation (p. 28-34)	○	○	○

2a) If you indicated above that any of the Phase 2 sections "need modification" or should be "removed", please explain below.

2b) Please provide any additional comments or suggestions for the Phase 2 guide content.

Guide Phase 3: Implementing, monitoring, and maintaining our program

3)

	Please select one statement per row		
	**Necessary: keep, as is**	**Necessary: needs modification** *(please explain below)*	**Not necessary: remove** *(please explain below)*
D - Launch the program (p. 37-38)	○	○	○
D - Celebrate the launch (p. 38)	○	○	○
E - Develop an evaluation plan; monitoring program delivery and use (p. 39-41)	○	○	○
F - Assess participant and program outcomes (p. 42-46)	○	○	○
G - Ensure continued operation of the program and respond to change (p. 46-48)	○	○	○

3a) If you indicated above that any of the Phase 3 sections "need modification" or should be "removed", please explain below.

3b) Please provide any additional comments or suggestions for the Phase 3 guide content.

PART B: INDIVIDUAL TOOLS AND RESOURCES

For each of the tools and resources listed below, **select one statement** which best describes your impression. Please provide comments or suggestions for modifications where you think it is necessary. There is space for your input at the end of the section.

Stroke Recovery in Motion Planner Tools and Resources

4)

	Please select one statement per row		
	**Necessary: keep, as is**	**Necessary: needs modification** *(please explain below)*	**Not necessary: remove** *(please explain below)*
Implementation Planning Roadmap (Phase 123 diagram, p. 53)	○	○	○
Project Charter: Template (p. 54)	○	○	○
Declaration of Conflict of Interest: Sample Disclosure (p. 55-57)	○	○	○
Community (Environmental) Scan: Worksheet (p. 58)	○	○	○
Community/Environmental Readiness: Worksheet (p. 59)	○	○	○
Sample Questions to Assess Community Readiness (p. 60-62)	○	○	○
Feasibility, Applicability, Acceptability: Checklist (p. 63-65)	○	○	○
Budget Planning Worksheet: Sample (p. 66-68)	○	○	○
Preparing the Business Case: Template (p. 69-70)	○	○	○
Implementation Work plan: Template (p. 71-73)	○	○	○
Identification of Barriers and Drivers: Template for Solution Building (p. 74-77)	○	○	○

	Please select one statement per row		
	**Necessary: keep, as is**	**Necessary: needs modification** *(please explain below)*	**Not necessary: remove** *(please explain below)*
Medical Authorization: Fit for Function Program Sample (p. 78-79)	○	○	○
Recreation Centre Readiness Checklist: TIME Program Sample (p. 80-81)	○	○	○
Participant Fitness Progress Log: Fit for Function excerpt (p. 82-83)	○	○	○
Program Sustainability Assessment Tool (p. 84-87)	○	○	○
Glossary (p. 88-91)	○	○	○
Appendix: The Knowledge to Action Cycle (p. 92-93)	○	○	○
Appendix: Decision-Making (p. 94-96)	○	○	○
Appendix: FAME, Fit for Function, TIME and HWE exercise program information (p. 97-103)	○	○	○
Appendix: Defining Indicators (p. 104)	○	○	○
Appendix: Outcome Measures (p. 105-107)	○	○	○
Appendix: Short Physical Performance Battery from FAME exercise program (p. 108)	○	○	○
Appendix: Sample Program Fidelity Checklists from FAME, Fit for Function, and TIME programs (p. 109-114)	○	○	○
Bibliography (p. 115)	○	○	○

4a) If you indicated above that any of the tools or resources in the Planner "need modification" or should be "removed", please explain below.

4b) Please provide any additional comments or suggestions for the tools or resources in the Planner.

PART C: FORMAT AND PRESENTATION

Please **select one statement** (on the scale between Strongly Agree to Strongly Disagree) to rate your impression of the usability of the Planner.

The Stroke Recovery in Motion planning material...
5)

	Please select one statement per row				
	Strongly Agree	Agree	Neither Agree or Disagree	Disagree	Strongly Disagree
is well organized	○	○	○	○	○
is written in plain language, easy to read and understand	○	○	○	○	○
clearly presents the planning process	○	○	○	○	○
clearly presents the research evidence for exercise	○	○	○	○	○
provides realistic, useful planning examples, tips, and stakeholder perspectives	○	○	○	○	○
includes helpful illustrations, pictures	○	○	○	○	○
the Phase 1-2-3 'Progress Checks' are helpful	○	○	○	○	○
provides easy access to worksheets, tools, templates and added resources	○	○	○	○	○
is user-friendly and easy to navigate	○	○	○	○	○
presents the right amount of information	○	○	○	○	○
I would prefer to use an on-line, web-based, interactive version vs. a paper version of the Planner	○	○	○	○	○

5a) Comments:

PART D: VALUE OF THIS RESOURCE FOR ASSISTING COMMUNITY PROGRAM PLANNERS

The Stroke Recovery in Motion Planner ...
6)

	Please select one statement per row				
	**Strongly Agree**	**Agree**	**Neither Agree or Disagree**	**Disagree**	**Strongly Disagree**
addresses the key factors to consider when planning an exercise program for people with stroke	○	○	○	○	○
uses a planning model supported by research	○	○	○	○	○
will help us make decisions informed by evidence	○	○	○	○	○
conflicts with our usual approach to planning	○	○	○	○	○
is compatible with our usual approach to planning	○	○	○	○	○
will improve our usual approach to planning	○	○	○	○	○
will require changes to our usual approach to planning	○	○	○	○	○
is suitable for most program planning scenarios	○	○	○	○	○
could be applied to planning for other programs	○	○	○	○	○
will meet the needs of community planners	○	○	○	○	○
will improve the sustainability of our program	○	○	○	○	○
will improve program outcomes	○	○	○	○	○

6a) Comments:

PART E: GENERAL IMPRESSIONS

Please comment on the following:

7) What I like most about the planning approach and/or materials:

8) What I like least about the planning approach and/or materials:

9) What is missing from the Planner:

10) My recommendations for improving the Planner:

11)	Please select one statement per row				
	Very likely	Likely	Neutral	Unlikely	Very unlikely
Hypothetically, if you were thinking about future program planning, how likely is it that you would use the Planner?	○	○	○	○	○
How likely is it that you would provide the Planner to other colleagues to support program planning?	○	○	○	○	○

PART F: YOUR COMMUNITY AND EXERCISE PROGRAM PLANS

Please tell us about your community, your role / experience, and any potential plans for introducing a community-based exercise program for people with stroke.

12) Location of your community:

Choose one of the following answers:

- ○ Alberta
- ○ British Columbia
- ○ Manitoba
- ○ New Brunswick
- ○ Newfoundland and Labrador
- ○ North West Territories
- ○ Nova Scotia
- ○ Nunavut
- ○ Ontario
- ○ Prince Edward Island
- ○ Quebec
- ○ Saskatchewan
- ○ Yukon Territory
- ○ Other – please describe:____

13) Population density of your community:

Choose one of the following answers:

- ○ Rural or mostly rural
- ○ Urban or mostly urban
- ○ Other - please describe:

14) Please enter the first three characters of your community's postal code:______________________

15) Size of your community:

Choose one of the following answers:

- ○ less than 5,000
- ○ 5,000 - 9,999
- ○ 10,000 - 24,999
- ○ 25,000 - 50,000
- ○ more than 50,000

16) When are you considering offering a community-based exercise program for people living with stroke?

Choose one of the following answers:

- ○ In less than 6 months
- ○ In 6 months to 1 year
- ○ In over a year
- ○ We currently do not intend to plan a program

17) If you were to proceed with offering an exercise program, indicate the type of organization / facility that you would use to host/deliver the program:

Check all that apply

- ☐ Community Recreation Centre (e.g. Public, municipal)
- ☐ YMCA
- ☐ Community health centre
- ☐ Seniors recreation centre
- ☐ Physiotherapy clinic
- ☐ Nursing home
- ☐ Retirement residence
- ☐ Private gym
- ☐ Other - please describe:

PART G: DEMOGRAPHICS & YOUR EXPERIENCE

The Planner was designed to help users understand evidence-based decision-making for program planning and the requirements for adapted and specialized programs. We are interested in learning about your experience with this approach to planning.

18) What is your gender?

Please choose one of the following answers:

- ○ Male
- ○ Female
- ○ Gender fluid
- ○ Other
- ○ Prefer not to answer

19) How many years of experience do you have in community program planning and/or delivery?

______________________years

20) Do you currently work for an organization that serves the community?

Choose one of the following answers:

- ○ Yes → answer question 20a
- ○ No → skip to question 21

20a) If yes, what type of organization do you currently work for?

Choose one of the following answers:

- ☐ Regional or provincial health authority
- ☐ Municipality
- ☐ Hospital
- ☐ Outpatient clinic
- ☐ Community clinic
- ☐ Patient advocacy organization
- ☐ Other – please describe:____

21) What is your current role?

Choose one of the following answers:

- ○ Provider Agency Administration
- ○ Program Manager/Coordinator
- ○ Fitness Instructor
- ○ Physiotherapist
- ○ Physician
- ○ Nurse
- ○ A person living with stroke or a caregiver of person living with stroke
- ○ Volunteer
- ○ Other - please describe:____

22) What is your experience in *planning an adapted or specialized fitness* program?

Choose one of the following answers:

- ○ **No experience** planning adapted or specialized fitness programs
- ○ **Previous experience** planning adapted or specialized fitness programs

Please comment on your choice here if needed:

23) What is your experience in *delivering* this type of program?

Choose one of the following answers:

- ○ **No experience** delivering adapted or specialized fitness programs
- ○ **Previous experience** delivering adapted or specialized fitness programs
- ○ **Not applicable** – my role is not to deliver fitness programs

Please comment on your choice here if needed:

24) Please rate your current confidence in *planning* an adapted or specialized fitness program:

Choose one of the following answers:

- ○ Not at all confident
- ○ Slightly confident
- ○ Moderately confident
- ○ Very confident
- ○ Extremely confident

25) Has reading the Planner had any effect on your confidence in your ability to plan an adapted or specialized fitness program?

Choose one of the following answers:

- ○ Yes, reading the Planner *increased* my confidence
- ○ Yes, reading the Planner *decreased* my confidence
- ○ No, reading the Planner had *no effect* on my confidence

26) How knowledgeable are you of how to use evidence to inform decision-making in program planning?
Choose one of the following answers:

- ○ Not at all knowledgeable
- ○ Slightly knowledgeable
- ○ Moderately knowledgeable
- ○ Very knowledgeable
- ○ Extremely knowledgeable

27) Has reading the Planner improved your knowledge of how to use evidence to inform decision-making in program planning?
Choose one of the following answers:

- ○ Yes, reading the Planner *increased* my knowledge
- ○ No, reading the Planner had *no effect* on my knowledge

28) Is there anything else you would like to tell us?

Thank you for your input!

PAST Program Planner Questionnaire
PART A: CONTENT OF GUIDE

Thinking about your past experience planning an exercise program and your review of the planning material, for each of the planner sections listed below:

- First, please indicate whether you considered the following issues during your own previous exercise program planning process by selecting “yes” or “no”;
- Second, please indicate whether you think the section of the Planner is “necessary”, “requires modification”, or should be “removed.”

Please provide comments or suggestions for modifications where you think it is necessary. There is space for your input at the end of each section.

Guide Phase 1: Understanding our needs, population, and resources

1)

	**When my team planned an exercise program, we considered this:**		**I think this section of the Planner is:**		
	**Yes**	**No**	**Necessary: keep, as is**	**Necessary: needs modification** *(please explain below)*	**Not necessary: remove** *(please explain below)*
A1 - Explore the "call to action"; thinking about starting a program (p. 2-3)	○	○	○	○	○
A2 - Involve the community; identify key partners (p. 3-5)	○	○	○	○	○
A2 - Develop your team’s terms of reference; creating a project charter (p. 6)	○	○	○	○	○
A3 - Form consensus about the importance of exercise for people with stroke (p. 7-8)	○	○	○	○	○

A3 – Assess features of exercise programs designed for people with stroke (p. 8-11)	○	○	○	○	○
A4 - Conduct community assessment (p. 12)	○	○	○	○	○
A5 - Choose a program that is a good fit for your community (p. 13-14)	○	○	○	○	○
A5 – Consider cost implications (p. 14-16)	○	○	○	○	○
Figure 3 - Decision making: should we and can we proceed with an exercise program here? (p. 17)	○	○	○	○	○
A6 - Prepare your business case (p. 18)	○	○	○	○	○
A6 - Prepare your implementation work plan (p. 19)	○	○	○	○	○
A7 - Achieve agreement to proceed (or not) (p. 19)	○	○	○	○	○

1a) If you indicated above that any of the Phase 1 sections "need modification" or should be "removed", please explain below.

1b) Please provide any additional comments or suggestions for the Phase 1 guide content.

Guide Phase 2: Building solutions that work for us

2)

	**When my team planned an exercise program, we considered this:**		**I think this section of the Planner is**		
	**Yes**	**No**	**Necessary: keep, as is**	**Necessary: needs modification** *(please explain below)*	**Not necessary: remove** *(please explain below)*
B - Assess barriers and drivers to program implementation (p. 22-28)	○	○	○	○	○
C - Address identified challenges to program implementation (p. 28-34)	○	○	○	○	○

2a) If you indicated above that any of the Phase 2 sections "need modification" or should be "removed", please explain below.

2b) Please provide any additional comments or suggestions for the Phase 2 guide content.

Guide Phase 3: Implementing, monitoring, and maintaining our program

3)

	When my team planned an exercise program, we consider this:		I think this section of the planner is		
	**Yes**	**No**	**Necessary: keep, as is**	**Necessary: needs modification** *(please explain below)*	**Not necessary: remove** *(please explain below)*
D - Launch the program (p. 37-38)	○	○	○	○	○
D - Celebrate the launch (p. 38)	○	○	○	○	○
E - Develop an evaluation plan; monitoring program delivery and use (p. 39-41)	○	○	○	○	○
F - Assess participant and program outcomes (p. 42-46)	○	○	○	○	○
G - Ensure continued operation of the program and respond to change (p. 46-48)	○	○	○	○	○

3a) If you indicated above that any of the Phase 3 sections "need modification" or should be "removed", please explain below.

3b) Please provide any additional comments or suggestions for the Phase 3 guide content.

PART B: INDIVIDUAL TOOLS AND RESOURCES

For each of the tools and resources listed below, **select one statement** which best describes your impression. Please provide comments or suggestions for modifications where you think it is necessary. There is space for your input at the end of the section.

Stroke Recovery in Motion Planner Tools and Resources

4)

	Please select one statement per row		
	**Necessary: keep, as is**	**Necessary: needs modification** *(please explain below)*	**Not necessary: remove** *(please explain below)*
Implementation Planning Roadmap (Phase 123 diagram, p. 53)	○	○	○
Project Charter: Template (p. 54)	○	○	○
Declaration of Conflict of Interest: Sample Disclosure (p. 55-57)	○	○	○
Community (Environmental) Scan: Worksheet (p. 58)	○	○	○
Community/Environmental Readiness: Worksheet (p. 59)	○	○	○
Sample Questions to Assess Community Readiness (p. 60-62)	○	○	○
Feasibility, Applicability, Acceptability: Checklist (p. 63-65)	○	○	○
Budget Planning Worksheet: Sample (p. 66-68)	○	○	○
Preparing the Business Case: Template (p. 69-70)	○	○	○
Implementation Work plan: Template (p. 71-73)	○	○	○

	Please select one statement per row		
	**Necessary: keep, as is**	**Necessary: needs modification** *(please explain below)*	**Not necessary: remove** *(please explain below)*
Identification of Barriers and Drivers: Template for Solution Building (p. 74-77)	○	○	○
Medical Authorization: Fit for Function Program Sample (p. 78-79)	○	○	○
Recreation Centre Readiness Checklist: TIME Program Sample (p. 80-81)	○	○	○
Participant Fitness Progress Log: Fit for Function excerpt (p. 82-83)	○	○	○
Program Sustainability Assessment Tool (p. 84-87)	○	○	○
Glossary (p. 88-91)	○	○	○
Appendix: The Knowledge to Action Cycle (p. 92-93)	○	○	○
Appendix: Decision-Making (p. 94-96)	○	○	○
Appendix: FAME, Fit for Function, TIME and HWE exercise program information (p. 97-103)	○	○	○
Appendix: Defining Indicators (p. 104)	○	○	○
Appendix: Outcome Measures (p. 105-107)	○	○	○
Appendix: Short Physical Performance Battery from FAME exercise program (p. 108)	○	○	○
Appendix: Sample Program Fidelity Checklists from FAME, Fit for Function, and TIME programs (p. 109-114)	○	○	○
Bibliography (p. 115)	○	○	○

4a) If you indicated above that any of the tools or resources in the Planner “need modification” or should be “removed”, please explain below.

4b) Please provide any additional comments or suggestions for the tools or resources in the Planner.

PART C: FORMAT AND PRESENTATION

Please **select one statement** (on the scale between Strongly Agree to Strongly Disagree) to rate your impression of the usability of the Planner.

The Stroke Recovery in Motion planning material...

**5)**	**Please select one statement per row**				
	**Strongly Agree**	**Agree**	**Neither Agree or Disagree**	**Disagree**	**Strongly Disagree**
is well organized	○	○	○	○	○
is written in plain language, easy to read and understand	○	○	○	○	○
clearly presents the planning process	○	○	○	○	○
clearly presents the research evidence for exercise	○	○	○	○	○
provides realistic, useful planning examples, tips, and stakeholder perspectives	○	○	○	○	○
includes helpful illustrations, pictures	○	○	○	○	○
the Phase 1-2-3 'Progress Checks' are helpful.	○	○	○	○	○
provides easy access to worksheets, tools, templates and added resources	○	○	○	○	○
is user-friendly and easy to navigate	○	○	○	○	○
presents the right amount of information	○	○	○	○	○
I would prefer to use an on-line, web-based, interactive version vs. a paper version of the Planner	○	○	○	○	○

5a) Comments:

PART D: VALUE OF THIS RESOURCE FOR ASSISTING COMMUNITY PROGRAM PLANNERS

The Stroke Recovery in Motion Planner ...

6)	Please select one statement per row				
	**Strongly Agree**	**Agree**	**Neither Agree or Disagree**	**Disagree**	**Strongly Disagree**
addresses the key factors to consider when planning an exercise program for people with stroke	○	○	○	○	○
uses a planning model supported by research	○	○	○	○	○
will help planning teams make decisions informed by evidence	○	○	○	○	○
conflicts with our usual approach to planning	○	○	○	○	○
is compatible with our usual approach to planning	○	○	○	○	○
could improve our usual approach to planning	○	○	○	○	○
would require changes to our usual approach to planning	○	○	○	○	○
is suitable for most program planning scenarios	○	○	○	○	○
could be applied to planning for other programs	○	○	○	○	○
will meet the needs of community planners	○	○	○	○	○
could improve the sustainability of programs	○	○	○	○	○
could improve program outcomes	○	○	○	○	○

6a) Comments:

PART E: GENERAL IMPRESSIONS

7) What I like most about the planning approach and/or materials:

8) What I like least about the planning approach and/or materials:

9) What is missing from the Planner:

10) My recommendations for improving the Planner:

11)

	Please select one statement per row				
	**Very likely**	**Likely**	**Neutral**	**Unlikely**	**Very unlikely**
Hypothetically, if you were thinking about future program planning, how likely is it that you would use the Planner?	○	○	○	○	○
How likely is it that you would provide the Planner to other colleagues to support program planning?	○	○	○	○	○

PART F: YOUR COMMUNITY AND EXERCISE PROGRAM

Please tell us about your community, your role / experience, and your previous planning / implementation of a community-based exercise program for people with stroke.

12) Location of community where exercise program was planned/implemented:
Choose one of the following answers:

- ○ Alberta
- ○ British Columbia
- ○ Manitoba
- ○ New Brunswick
- ○ Newfoundland and Labrador
- ○ North West Territories
- ○ Nova Scotia
- ○ Nunavut
- ○ Ontario
- ○ Prince Edward Island
- ○ Quebec
- ○ Saskatchewan
- ○ Yukon Territory
- ○ Other:____

13) Population density of community where exercise program was planned/implemented:
Choose one of the following answers:

- ○ Rural or mostly rural
- ○ Urban or mostly urban
- ○ Other - please describe:

14) Please enter the first three characters of your organization's postal code:____________________

15) Size of community where exercise program was planned/implemented

Choose one of the following answers:

- ○ less than 5,000
- ○ 5,000 - 9,999
- ○ 10,000 - 24,999
- ○ 25,000 - 50,000
- ○ more than 50,000

16) Did you implement a community-based exercise program for people with stroke in your community?

Choose one of the following answers:

- ○ Yes → answer questions 17-24 below, as applicable
- ○ No → skip directly to questions 25 - 26 (in grey box on page 17)

17) If YES, is the program still running?

Choose one of the following answers:

- ○ Yes → go to question 18
- ○ No → skip to questions 19 and 20

18) To date, how many cycles of the program have you run

19) If the program is not still running, how many cycles did you run before stopping it?

20) If the program is not still running, please explain the decision to stop the program.

21) Please provide the name and a brief description of the program you offered:

22) What was your enrolment in the program?

If you had multiple sessions, please indicate the minimum and maximum enrolment you saw.

____________(minimum)	____________(maximum)

23) Indicate the type of organization / facility in which you offered the exercise program:
Check all that apply:

- ☐ Community Recreation Centre (e.g. public, municipal)
- ☐ YMCA
- ☐ Community Health Centre
- ☐ Seniors Recreation Centre
- ☐ Physiotherapy Clinic
- ☐ Nursing Home
- ☐ Retirement Residence
- ☐ Private Gym
- ☐ Other - please describe:____

24) Approximately how many months was it from the time your team had the idea to start planning an exercise program for people living to stroke to the time you launched the program?

_______________months

If you did NOT implement a community-based exercise program for people with stroke in your community *(i.e., you answered NO to question 16 above)*, **please answer questions 25 and 26 below.**

25) Please explain why the program was never implemented in your community.

26) Approximately how many months was it from the time your team had the idea to start planning an exercise program for people living to stroke to the time you made the decision not to launch a program?

________________months

27) Did your planning team use a formal process or framework to guide your exercise program planning?

- ○ Yes
- ○ No

Please explain:

28) Who was on your planning team?

Check all that apply:

- ☐ Members from regional health authority
- ☐ Members from municipal parks and recreation department (managers, coordinators)
- ☐ Leaders from the municipality
- ☐ Members from patient advocacy groups
- ☐ Fitness instructor(s)
- ☐ Physiotherapist(s)
- ☐ Physician(s)
- ☐ Person(s) living with stroke
- ☐ Caregiver(s) of person living with stroke
- ☐ Other, please specify:____

29) Did your planning team take any steps that were not covered in the Planner?

Choose one of the following answers:

- ○ Yes → answer question 29a
- ○ No → skip to question 30

29a) If yes, please briefly describe the steps you took that were not covered in the Planner.

30) Do you have any additional tools / resources that you think should be included in the Planner?

Choose one of the following answers:

- ○ Yes→ answer question 30a
- ○ No → skip to question 31 on the next page

30a) If yes, please describe the additional tools / resources that you think should be included in the Planner:

31) If you had access to the Planner when you previously planned an exercise program, would the Planner have improved…

	Please select one statement		
	**Yes**	**No**	**Uncertain**
the planning process?	○	○	○
your ability to make an informed decision to proceed with program implementation (or not)?	○	○	○
the sustainability of the program?	○	○	○

31a) Please provide any additional comments on how the Planner may have improved your previous planning process (or not):

PART G: DEMOGRAPHICS & YOUR EXPERIENCE

The Planner was designed to help users understand evidence-based decision-making for program planning and the requirements for adapted and specialized programs. We are interested in learning about your experience with this approach to planning.

32) What is your gender?

Please choose one of the following answers:

- ○ Male
- ○ Female
- ○ Gender fluid
- ○ Other
- ○ Prefer not to answer

33) How many years of experience do you have in community program planning and/or delivery?

_______________________ years

34) What was your individual role in planning or delivering the exercise program?

Choose one of the following answers:

- ○ Provider Agency Administration
- ○ Program Manager/Coordinator
- ○ Fitness Instructor
- ○ Health Partner
- ○ Sponsoring Partner
- ○ Participant
- ○ Other - please describe:

35) What was your experience in *planning* this type of program?

Choose one of the following answers:

- ○ This was my **first time** planning this type of program
- ○ I had **previous experience** planning adapted or specialized fitness programs

Please comment on your choice here if needed:

36) What was your experience in *delivering* this type of program?

Choose one of the following answers:

- ○ This was my **first time** delivering this type of program
- ○ I had **previous experience** delivering adapted or specialized fitness programs
- ○ Not applicable – I was not involved in delivering the program

Please comment on your choice here if needed:

37) Please rate your current confidence in planning an adapted or specialized fitness program:

Choose one of the following answers:

- ○ Not at all confident
- ○ Slightly confident
- ○ Moderately confident
- ○ Very confident
- ○ Extremely confident

38) Has reading the Planner had any effect on your confidence in your ability to plan an adapted or specialized fitness program?

Choose one of the following answers:

- ○ Yes, reading the Planner *increased* my confidence
- ○ Yes, reading the Planner *decreased* my confidence
- ○ No, reading the Planner had *no effect* on my confidence

39) How knowledgeable are you of how to use evidence to inform decision-making in program planning?

Choose one of the following answers:

- ○ Not at all knowledgeable
- ○ Slightly knowledgeable
- ○ Moderately knowledgeable
- ○ Very knowledgeable
- ○ Extremely knowledgeable

40) Has reading the Planner improved your knowledge of how to use evidence to inform decision-making in program planning?

Choose one of the following answers:

- ○ Yes, reading the Planner *increased* my knowledge
- ○ No, reading the Planner had *no effect* on my knowledge

41) Is there anything else you would like to tell us?

Thank you for your input!
