# Supplementary material for: The Stroke Recovery in Motion Implementation Planner: Mixed Methods User Evaluation
Source: JMIR Form Res. 2022 Jul 29;6(7):e37189. doi: 10.2196/37189 (PMC9377478; doi:10.2196/37189)
Supplement: Multimedia Appendix 3 [file formative_v6i7e37189_app3.pdf]

Multimedia Appendix – Semi-Structured Interview/Focus Group Guides

This is a Multimedia Appendix to a full manuscript published in the JMIR Form Res. For full copyright and citation information see http://dx.doi.org/10.2196/37189

Baseline Questions for *Current* program planners

1. Let's start by talking about usual processes for planning programs at your organization.
   - What is the usual planning process? Is there a formal framework or process that you follow?
     - Who initiates program planning at your organization?
     - What is the motivation - key factors/key moment - that triggers contemplation of a new program?
     - How do you set priorities? How are the decisions made? Who makes the decisions? What is the approval process? How do you allocate limited resources?
     - Which populations need to be included to create critical mass? (i.e., how many participants do you need to move forward? How did you decide?)

2. Now let's talk more specifically about implementing a community-based exercise program for people living with stroke.
   - Tell me how it came about that your community started thinking about implementing a community-based exercise program for people living with stroke?
   - Had any planning work been done before you enrolled in this study? If yes:
     - Tell me about what work had been done. Walk me through the steps you took.
       - When did your planning start?
       - Who was involved?
       - Are there any previously completed planning documents you could share with us?
     - How did the steps you previously took compare to the Planner?
       - If completed a step in the Planner before study enrollment – how close is what we suggested to what you actually did?
       - Did you take any steps not covered in the Planner? Did we miss anything?
     - If you had access to the Planner before, would you have approached things differently?

3. Let's now turn to the Planner and its tools and your initial impressions.
   - Could you start by telling me your initial reaction to the planning process described in the Planner?
     - Does it make sense to you?
     - Are the phases the right phases?
     - Are the steps the right steps?
   - How similar or dissimilar is the process to your current planning process?
   - How feasible do you think the process will be to follow?
   - What do you think will be the challenges of following the Planner? How might you overcome those challenges?
   - What do you think of the Planner in general?
     - What do you like or dislike about it?
     - How complete is it?
     - What do you think of the tools?

4. Now let’s talk about some of the recommended modifications to the Planner and tools.
   [If participant completed survey]
   - Thank you for completing the survey. I have looked over your responses and want to talk more in depth about some of your feedback. Let’s talk about some of the specific items that you recommended should be changed or removed in your survey.
     - You identified that section(s) X needed modification….[probe as needed]
     - You identified hat section(s) X should be removed…..[probe as needed]

5. Now let’s talk about your planning team – including how it was formed and how you are working together at this point.
   - Tell me who is on the planning team?
   - Who would be the person(s) responsible for leading program planning (local champion)?
   - How did you become engaged with this planning team?
   - Tell me about your role and responsibilities on the team.
   - As you have been building the planning team, what has been working well to engage the team members? Have you encountered any challenges to date?
   - Who else have you identified as key stakeholders? What do you anticipate the role of these other stakeholders will be?
     i. Examples might include: any other people in the community with an interest in the success of the program, funders, people living with stroke, delivery personnel, people in the referral network, caregivers, etc.

6. Other comments
   - Do you have any other comments on the Planner?
   - Do you have any other comments on your team’s plans for implementing a community-based exercise program for people living with stroke?

Monitoring Questions for *Current* program planners

1. Tell me about what your team has worked on since we last talked.

2. Have you been referring to the planner? If so, which sections have you been working through? How did it go?

3. Have you completed any of the tools? If so, who completed them? If so, can we have a copy of completed tools?

4. Did you use any other tools or resources? If so, what were they? Is this something we should consider adding to the planner?

5. What is your key issue / most pressing need / biggest challenge right now? Do you need help with anything? Are you getting the help you need?

6. Have there been any positive developments since we last spoke? Biggest success? What is helping the most?

7. What are your next steps?

End-of-study Questions for *Current* program planners

1. How did using the Planner contribute to your experience planning a community-based exercise program?

2. Reflecting over your past few months using the Planner:
    - What parts of the Planner were the most helpful?
    - Are there steps you followed but in hindsight don't think were helpful?
    - Is there anything you felt was missing from the Planner?

3. While the formal SRiM study is ending, we are wondering if you will continue to use the Planner as you continue your work to plan and deliver a community-based program for people living with stroke? Why or why not.

4. Would you recommend the Planner to others? If so, who?

5. What would be your advice for another team on how to use the Planner?

6. During our previous calls, you indicated that you completed [*names of SRiM tools*] and/or used your own tools [*names of own tools*]. As I had mentioned at the start of the study, we are interested in collecting these documents from our participating sites so we can learn how real-world teams use the Planner tools and other resources. Would you be willing to share your completed tools with us? You could remove any identifying information before sending them to me.

7. Is there anything else you wanted to share?

Follow-up Questions for *Future* program planners

1. Could you start by telling me your initial reaction to the planning process described in the Planner?
   - Does it make sense to you?
   - Are the 3 phases the right phases?
     - Understanding our needs, population and resources;
     - Building solutions that work for us;
     - Implementing, monitoring and maintaining our exercise program.
   - Are the steps under each phase the right steps?

2. Tell me what you liked about the Planner? What did you dislike about the Planner?

3. Thank you for completing the survey. I have looked over your responses and want to talk more in depth about some of your feedback. Let's talk about some of the specific items that you recommended should be changed or removed.
   - You identified that section(s) X needed modification….[probe as needed]
   - You identified that section(s) X should be removed…..[probe as needed]

4. Would you use the Planner in the future to facilitate program planning? How come?
   - Would you recommend the Planner to others? How come and to who?

5. How feasible do you think the process in the Planner would be for planning teams to follow?
   - What do you think would be the challenges for teams following the Planner?
   - How might teams overcome those challenges?

6. Do you have any other comments on the Planner? Do you have any other comments on your team's experience planning and implementing a community-based exercise program for people living with stroke?

Questions on past experience for *Past* program planners

1. Tell me how it came about that your community started thinking about implementing a community-based exercise program for people living with stroke?
    a. When did your planning start?
    b. What was the motivation (key factors/key moment) that triggered contemplation of this new program?

2. Who were the members of your planning team? Who were your key stakeholders and partners?
    a. What were their roles and responsibilities?
    b. How did roles and responsibilities change over time?
    c. Who was the person(s) responsible for leading program planning (local champion)? What attributes made that person an (in)effective lead/champion?
    d. Looking back on the experience, were there any key stakeholders or partners that should have been on the planning team that weren't? Was there anyone on the team that maybe didn't need to be?

3. Tell me about the participation level of your team members – how much time and effort was contributed by the different planning team members throughout the planning process?
    a. Do you recall about how many meetings did you have?
    b. How did you decide how often to have meetings?
        - e.g., did you book regular meetings or did you only meet as needed? Did the frequency of meetings change depending on what phase of planning you were in?
    c. How did you decide who was invited to the meetings?
        - e.g., was every team member invited to every meeting? Or did you only invite members who were directly involved in the activities being discussed?
    d. Do you recall the purposes of these meeting? For example, what decisions had to be made or what preparations had to be discussed?

4. As you built the planning team, what worked well to engage the team members? Did you encounter any challenges? How did you deal with the challenges?
    a. Is there anything you would have done differently to improve how your team worked together through the planning process?
    b. Were you satisfied with your engagement on the team? Were you satisfied with your role, responsibilities and contributions?
    c. With the engagement of other team members?

5. If I wanted to start a community-based exercise program for people with stroke, based on your past experience planning this type of program, what steps would you recommend?
    a. Are there any formal processes or frameworks I should follow?
    b. Are there specific planning tasks and activities I should consider? For example, how would I proceed with:
        - Completing a community needs assessment?
        - Preparing a business case?
        - Choosing a program? Tailoring the program or delivery approach? How?
        - Addressing space? Equipment? Budget? Staffing? Training? Accessibility to program/transportation?
    c. Are there any tools or resources you would recommend?

d. What do you anticipate would be the top three barriers I might face?
e. What might be the top three drivers?
f. What are your top 3 tips for a new planning team?

6. (A) If the program was never launched:
   a. Why was the program not launched?
   b. What helped you make this decision?
   c. How satisfied are you that this was the right decision at that time?

7. (B) If the program was launched:
   a. To date, how many cycles of the program have you run?
   b. Overall, how successful was the implementation of the exercise program? Please explain.
   c. What are the key factors that influenced your success or lack of success?
   d. What were your indicators for success? How well do you think you met them?

   6B.1 *If the program was sustained:
   - What are the key factors that influenced your success sustaining the program?
     - Were any of the following factors key to your sustainability: (1) funding/costs, (2) compatibility with your organizations missions, (3) staffing, and (4) accessibility to program/transportation?
   - Have you made changes to the program over time? How come?

   6B.2 *If the program was not sustained:
   - What were the key factors that led to your decision to stop offering the program?
     - Were any of the following contributing factors: (1) funding/costs, (2) compatibility with your organizations missions, (3) staffing, and (4) accessibility to program/transportation?
   - What helped make this decision to stop offering the program?
   - How satisfied are you that this was the right decision at that time?

8. Do you have any other comments on your team's experience planning and implementing a community-based exercise program for people living with stroke?

Follow-up Questions for *Past* program planners

1. Could you start by telling me your initial reaction to the planning process described in the Planner?
   - Does it make sense to you?
   - Are the 3 phases the right phases?
     - Understanding our needs, population and resources;
     - Building solutions that work for us;
     - Implementing, monitoring and maintaining our exercise program.
   - Are the steps the right steps?

2. Tell me what you liked about the Planner? What did you dislike about the Planner?

3. *[If participant completed survey]*

   Thank you for completing the survey. I have looked over your responses and want to talk more in depth about some of your feedback. Let's talk about some of the specific items that you recommended should be changed or removed.
   - You identified that section(s) X needed modification….[probe as needed]
   - You identified that section(s) X should be removed…..[probe as needed]

4. Thinking about your own experience planning an exercise program, how did the process you used compare to the steps described in the Planner?
   - How close is the planning process described in the planner to what you actually did?
   - Were there steps in the Planner you didn't take?
   - Did you take any steps not covered in the Planner? Did we miss anything?
   - Are there any tools or resources you used that are not included in the Planner that should be included?

5. If you had access to the Planner before you planned an exercise program, would you have approached things differently? How?

6. Would you use the Planner in the future to facilitate program planning? How come?
   - Would you recommend the Planner to others? How come and to who?

7. How feasible do you think the process in the Planner would be for planning teams to follow?
   - What do you think would be the challenges for teams following the Planner?
   - How might teams overcome those challenges?

8. Do you have any other comments on the Planner? Do you have any other comments on your team's experience planning and implementing a community-based exercise program for people living with stroke?
