# Supplementary material for: The Stroke Recovery in Motion Implementation Planner: Mixed Methods User Evaluation
Source: JMIR Form Res. 2022 Jul 29;6(7):e37189. doi: 10.2196/37189 (PMC9377478; doi:10.2196/37189)
Supplement: Multimedia Appendix 4 [file formative_v6i7e37189_app4.pdf]

Multimedia Appendix – Questionnaire data on Planner sections and tools by study group

This is a Multimedia Appendix to a full manuscript published in the JMIR Form Res. For full copyright and citation information see http://dx.doi.org/10.2196/37189

Note: Data highlighted when <75% of respondents in any given group selected "necessary: keep as is"

PLANNER

Planner Section	Response option	Group			All participants n (%)
		Current program planners n (%)	Future program planners n (%)	Past program planners n (%)	
Explore the "call to action"; thinking about starting a program	✓ Necessary: keep as is	15 (100)	9 (100)	12 (100)	36 (100)
	! Necessary: needs modification	0 (0)	0 (0)	0 (0)	0 (0)
	✘ Not necessary: remove	0 (0)	0 (0)	0 (0)	0 (0)
Involve the community; identify key partners	✓ Necessary: keep as is	14 (93.3)	8 (88.9)	12 (100)	34 (94.4)
	! Necessary: needs modification	1 (6.7)	1 (11.1)	0 (0)	2 (5.6)
	✘ Not necessary: remove	0 (0)	0 (0)	0 (0)	0 (0)
Develop your team's terms of reference; creating a project charter	✓ Necessary: keep as is	9 (60.0)	8 (88.9)	10 (83.3)	27 (75.0)
	! Necessary: needs modification	4 (26.7)	0 (0)	2 (16.7)	6 (16.7)
	✘ Not necessary: remove	2 (13.3)	1 (11.1)	0 (0)	3 (8.3)
Form consensus about the importance of exercise for people with stroke	✓ Necessary: keep as is	10 (66.7)	8 (88.9)	9 (75.0)	27 (75.0)
	! Necessary: needs modification	5 (33.3)	0 (0)	3 (25.0)	8 (22.2)
	✘ Not necessary: remove	0 (0)	1 (11.1)	0 (0)	1 (2.8)
Features of exercise programs designed for people with stroke	✓ Necessary: keep as is	13 (86.7)	5 (55.6)	12 (100)	30 (83.3)
	! Necessary: needs modification	2 (13.3)	3 (33.3)	0 (0)	5 (13.9)
	✘ Not necessary: remove	0 (0)	1 (11.1)	0 (0)	1 (2.8)
Conduct community assessment	✓ Necessary: keep as is	15 (100)	9 (100)	12 (100)	36 (100)
	! Necessary: needs modification	0 (0)	0 (0)	0 (0)	0 (0)
	✘ Not necessary: remove	0 (0)	0 (0)	0 (0)	0 (0)
Choose a program that is a good fit for your community	✓ Necessary: keep as is	14 (93.3)	7 (77.8)	11 (91.7)	32 (88.9)
	! Necessary: needs modification	1 (6.7)	2 (22.2)	1 (8.3)	4 (11.1)

Planner Section	Response option	Group			All participants n (%)
		Current program planners n (%)	Future program planners n (%)	Past program planners n (%)	
	✘ Not necessary: remove	0 (0)	0 (0)	0 (0)	0 (0)
Cost implications	✓ Necessary: keep as is	15 (100)	8 (88.9)	10 (83.3)	33 (91.7)
	! Necessary: needs modification	0 (0)	1 (11.1)	2 (16.7)	3 (8.3)
	✘ Not necessary: remove	0 (0)	0 (0)	0 (0)	0 (0)
Fig. 3 - Decision making: should we and can we proceed with an exercise program here?	✓ Necessary: keep as is	12 (80.0)	8 (88.9)	11 (91.7)	31 (86.1)
	! Necessary: needs modification	3 (20.0)	1 (11.1)	1 (8.3)	5 (13.9)
	✘ Not necessary: remove	0 (0)	0 (0)	0 (0)	0 (0)
Prepare your business case	✓ Necessary: keep as is	10 (66.7)	9 (100)	10 (83.3)	29 (80.6)
	! Necessary: needs modification	4 (26.7)	0 (0)	1 (8.3)	5 (13.9)
	✘ Not necessary: remove	1 (6.7)	0 (0)	1 (8.3)	2 (5.6)
Prepare your implementation work plan	✓ Necessary: keep as is	14 (93.3)	9 (100)	11 (91.7)	34 (94.4)
	! Necessary: needs modification	1 (6.7)	0 (0)	1 (8.3)	2 (5.6)
	✘ Not necessary: remove	0 (0)	0 (0)	0 (0)	0 (0)
Achieve agreement to proceed (or not)	✓ Necessary: keep as is	12 (80.0)	8 (88.9)	10 (83.3)	30 (83.3)
	! Necessary: needs modification	1 (6.7)	0 (0)	0 (0)	1 (2.8)
	✘ Not necessary: remove	2 (13.3)	1 (11.1)	2 (16.7)	5 (13.9)
Assess barriers and drivers to program implementation	✓ Necessary: keep as is	13 (86.7)	7 (77.8)	11 (91.7)	31 (86.1)
	! Necessary: needs modification	2 (13.3)	2 (22.2)	1 (8.3)	5 (13.9)
	✘ Not necessary: remove	0 (0)	0 (0)	0 (0)	0 (0)
Address identified challenges to program implementation	✓ Necessary: keep as is	12 (80.0)	4 (44.4)	12 (100)	28 (77.8)
	! Necessary: needs modification	3 (20.0)	5 (55.6)	0 (0)	8 (22.2)
	✘ Not necessary: remove	0 (0)	0 (0)	0 (0)	0 (0)
Launch the program	✓ Necessary: keep as is	15 (100)	8 (88.9)	11 (91.7)	34 (94.4)
	! Necessary: needs modification	0 (0)	1 (11.1)	1 (8.3)	2 (5.6)

Planner Section	Response option	Group			All participants n (%)
		Current program planners n (%)	Future program planners n (%)	Past program planners n (%)	
	✘ Not necessary: remove	0 (0)	0 (0)	0 (0)	0 (0)
Celebrate the launch	✓ Necessary: keep as is	11 (73.3)	8 (88.9)	10 (83.3)	29 (80.6)
	! Necessary: needs modification	3 (20.0)	0 (0)	2 (16.7)	5 (13.9)
	✘ Not necessary: remove	1 (6.7)	1 (11.1)	0 (0)	2 (5.6)
Develop an evaluation plan; monitoring program delivery and use	✓ Necessary: keep as is	12 (80.0)	7 (77.8)	9 (75.0)	28 (77.8)
	! Necessary: needs modification	3 (20.0)	2 (22.2)	3 (25.0)	8 (22.2)
	✘ Not necessary: remove	0 (0)	0 (0)	0 (0)	0 (0)
Assess participant and program outcomes	✓ Necessary: keep as is	13 (86.7)	8 (88.9)	9 (75.0)	30 (83.3)
	! Necessary: needs modification	2 (13.3)	1 (11.1)	3 (25.0)	6 (16.7)
	✘ Not necessary: remove	0 (0)	0 (0)	0 (0)	0 (0)
Ensure continued operation of the program and respond to change	✓ Necessary: keep as is	14 (93.3)	8 (88.9)	11 (91.7)	33 (91.7)
	! Necessary: needs modification	1 (6.7)	1 (11.1)	1 (8.3)	3 (8.3)
	✘ Not necessary: remove	0 (0)	0 (0)	0 (0)	0 (0)

PLANNER TOOLS AND RESOURCES

Planner Tool / Resource	Response option	Group			All Participants n (%)
		Current program planners n (%)	Future program planners n (%)	Past program planners n (%)	
Implementation Planning Roadmap	✓ Necessary: keep as is	11 (73.3)	8 (88.9)	12 (100)	31 (86.1)
	! Necessary: needs modification	4 (26.7)	1 (11.1)	0 (0)	5 (13.9)
	✘ Not necessary: remove	0 (0)	0 (0)	0 (0)	0 (0)
Project Charter: Template	✓ Necessary: keep as is	12 (80.0)	7 (77.8)	12 (100)	31 (86.1)
	! Necessary: needs modification	3 (20.0)	1 (11.1)	0 (0)	4 (11.1)
	✘ Not necessary: remove	0 (0)	1 (11.1)	0 (0)	1 (2.8)
Declaration of Conflict of	✓ Necessary: keep as is	14 (93.3)	8 (88.9)	9 (75.0)	31 (86.1)

Planner Tool / Resource	Response option	Group			All Participants n (%)
		Current program planners n (%)	Future program planners n (%)	Past program planners n (%)	
Interest: Sample Disclosure	! Necessary: needs modification	1 (6.7)	1 (11.1)	0 (0)	2 (5.6)
	✘ Not necessary: remove	0 (0)	0 (0)	3 (25.0)	3 (8.3)
Community (Environmental) Scan: Worksheet	✓ Necessary: keep as is	11 (73.3)	9 (100)	10 (83.3)	30 (83.3)
	! Necessary: needs modification	4 (26.7)	0 (0)	2 (16.7)	6 (16.7)
	✘ Not necessary: remove	0 (0)	0 (0)	0 (0)	0 (0)
Community/ Environmental Readiness: Worksheet	✓ Necessary: keep as is	10 (66.7)	7 (77.8)	11 (91.7)	28 (77.8)
	! Necessary: needs modification	4 (26.7)	0 (0)	1 (8.3)	5 (13.9)
	✘ Not necessary: remove	1 (6.7)	2 (22.2)	0 (0)	3 (8.3)
Sample Questions to Assess Community Readiness	✓ Necessary: keep as is	9 (60.0)	8 (88.9)	11 (91.7)	28 (77.8)
	! Necessary: needs modification	5 (33.3)	1 (11.1)	1 (8.3)	7 (19.4)
	✘ Not necessary: remove	1 (6.7)	0 (0)	0 (0)	1 (2.8)
Feasibility, Applicability, Acceptability: Checklist	✓ Necessary: keep as is	12 (80.0)	9 (100)	12 (100)	33 (91.7)
	! Necessary: needs modification	2 (13.3)	0 (0)	0 (0)	2 (5.6)
	✘ Not necessary: remove	1 (6.7)	0 (0)	0 (0)	1 (2.8)
Budget Planning Worksheet: Sample	✓ Necessary: keep as is	14 (93.3)	8 (88.9)	11 (91.7)	33 (91.7)
	! Necessary: needs modification	1 (6.7)	1 (11.1)	1 (8.3)	3 (8.3)
	✘ Not necessary: remove	0 (0)	0 (0)	0 (0)	0 (0)
Preparing the Business Case	✓ Necessary: keep as is	11 (73.3)	9 (100)	11 (91.7)	31 (86.1)
	! Necessary: needs modification	2 (13.3)	0 (0)	1 (8.3)	3 (8.3)
	✘ Not necessary: remove	2 (13.3)	0 (0)	0 (0)	2 (5.6)
Implementation Work plan: Template	✓ Necessary: keep as is	11 (73.3)	8 (88.9)	11 (91.7)	30 (83.3)
	! Necessary: needs modification	3 (20.0)	1 (11.1)	1 (8.3)	5 (13.9)
	✘ Not necessary: remove	1 (6.7)	0 (0)	0 (0)	1 (2.8)
Identification of Barriers and Drivers: Template for	✓ Necessary: keep as is	13 (86.7)	7 (77.8)	9 (75.0)	29 (80.6)
	! Necessary: needs modification	1 (6.7)	2 (22.2)	3 (25.0)	6 (16.7)

Planner Tool / Resource	Response option	Group			All Participants n (%)
		Current program planners n (%)	Future program planners n (%)	Past program planners n (%)	
Solution Building					
	✘ Not necessary: remove	1 (6.7)	0 (0)	0 (0)	1 (2.8)
Medical Authorization: Fit for Function Program Sample	✓ Necessary: keep as is	14 (93.3)	8 (88.9)	12 (100)	34 (94.4)
	! Necessary: needs modification	1 (6.7)	1 (11.1)	0 (0)	2 (5.6)
	✘ Not necessary: remove	0 (0)	0 (0)	0 (0)	0 (0)
Recreation Centre Readiness Checklist: TIME Program Sample	✓ Necessary: keep as is	14 (93.3)	7 (77.8)	12 (100)	33 (91.7)
	! Necessary: needs modification	0 (0)	1 (11.1)	0 (0)	1 (2.8)
	✘ Not necessary: remove	1 (6.7)	1 (11.1)	0 (0)	2 (5.6)
Participant Fitness Progress Log: Fit for Function excerpt	✓ Necessary: keep as is	12 (80.0)	6 (66.7)	11 (91.7)	29 (80.6)
	! Necessary: needs modification	2 (13.3)	2 (22.2)	1 (8.3)	5 (13.9)
	✘ Not necessary: remove	1 (6.7)	1 (11.1)	0 (0)	2 (5.6)
Program Sustainability Assessment Tool	✓ Necessary: keep as is	13 (86.7)	7 (77.8)	10 (83.3)	30 (83.3)
	! Necessary: needs modification	2 (13.3)	2 (22.2)	2 (16.7)	6 (16.7)
	✘ Not necessary: remove	0 (0)	0 (0)	0 (0)	0 (0)
Glossary	✓ Necessary: keep as is	15 (100)	8 (88.9)	12 (100)	35 (97.2)
	! Necessary: needs modification	0 (0)	1 (11.1)	0 (0)	1 (2.8)
	✘ Not necessary: remove	0 (0)	0 (0)	0 (0)	0 (0)
Appendix: The Knowledge to Action Cycle	✓ Necessary: keep as is	8 (53.3)	8 (88.9)	11 (91.7)	27 (75.0)
	! Necessary: needs modification	4 (26.7)	0 (0)	0 (0)	4 (11.1)
	✘ Not necessary: remove	3 (20.0)	1 (11.1)	1 (8.3)	5 (13.9)
Appendix: Decision-Making	✓ Necessary: keep as is	10 (66.7)	8 (88.9)	9 (75.0)	27 (75.0)
	! Necessary: needs modification	2 (13.3)	0 (0)	0 (0)	2 (5.6)
	✘ Not necessary: remove	3 (20.0)	1 (11.1)	3 (25.0)	7 (19.4)
Appendix: FAME, Fit for Function, TIME	✓ Necessary: keep as is	12 (80.0)	8 (88.9)	12 (100)	32 (88.9)

Planner Tool / Resource	Response option	Group			All Participants n (%)
		Current program planners n (%)	Future program planners n (%)	Past program planners n (%)	
and HWE exercise program information	! Necessary: needs modification	3 (20.0)	1 (11.1)	0 (0)	4 (11.1)
	✘ Not necessary: remove	0 (0)	0 (0)	0 (0)	0 (0)
Appendix: Defining Indicators	✓ Necessary: keep as is	12 (80.0)	8 (88.9)	11 (91.7)	31 (86.1)
	! Necessary: needs modification	2 (13.3)	0 (0)	1 (8.3)	3 (8.3)
	✘ Not necessary: remove	1 (6.7)	1 (11.1)	0 (0)	2 (5.6)
*Appendix: Outcome Measures	✓ Necessary: keep as is	11 (91.7)	8 (88.9)	10 (83.3)	29 (87.9)
	! Necessary: needs modification	1 (8.3)	1 (11.1)	2 (16.7)	4 (12.1)
	✘ Not necessary: remove	0 (0)	0 (0)	0 (0)	0 (0)
**Appendix: Short Physical Performance Battery from FAME exercise program	✓ Necessary: keep as is	14 (100)	7 (77.8)	12 (100)	33 (94.3)
	! Necessary: needs modification	0 (0)	2 (22.2)	0 (0)	2 (5.7)
	✘ Not necessary: remove	0 (0)	0 (0)	0 (0)	0 (0)
*Appendix: Sample Program Fidelity Checklists from FAME, Fit for Function, and TIME programs	✓ Necessary: keep as is	11 (91.7)	8 (88.9)	11 (91.7)	30 (90.9)
	! Necessary: needs modification	0 (0)	1 (11.1)	1 (8.3)	2 (6.1)
	✘ Not necessary: remove	1 (8.3)	0 (0)	0 (0)	1 (3.0)
**Bibliography	✓ Necessary: keep as is	14 (100)	9 (100)	12 (100)	35 (100)
	! Necessary: needs modification	0 (0)	0 (0)	0 (0)	0 (0)
	✘ Not necessary: remove	0 (0)	0 (0)	0 (0)	0 (0)

* *Data missing on 3 Current program planner participants due to completing an earlier version of the questionnaire*
***Data missing on 1 Current program planner participant due to skipping the survey question*
